# Comparison of the transmission efficiency and plague progression dynamics associated with two mechanisms by which fleas transmit *Yersinia pestis*

**DOI:** 10.1371/journal.ppat.1009092

**Published:** 2020-12-07

**Authors:** Christopher F. Bosio, Clayton O. Jarrett, Dana P. Scott, Jonathan Fintzi, B. Joseph Hinnebusch

**Affiliations:** 1 Laboratory of Bacteriology, Rocky Mountain Laboratories, National Institute of Allergy and Infectious Diseases, National Institutes of Health, Hamilton, Montana, United States of America; 2 Rocky Mountain Veterinary Branch, Rocky Mountain Laboratories, National Institute of Allergy and Infectious Diseases, National Institutes of Health, Hamilton, Montana, United States of America; 3 Biostatistics Research Branch, National Institute of Allergy and Infectious Diseases, National Institutes of Health, Rockville, Maryland, United States of America; Stanford University School of Medicine, UNITED STATES

## Abstract

*Yersinia pestis* can be transmitted by fleas during the first week after an infectious blood meal, termed early-phase or mass transmission, and again after *Y*. *pestis* forms a cohesive biofilm in the flea foregut that blocks normal blood feeding. We compared the transmission efficiency and the progression of infection after transmission by *Oropsylla montana* fleas at both stages. Fleas were allowed to feed on mice three days after an infectious blood meal to evaluate early-phase transmission, or after they had developed complete proventricular blockage. Transmission was variable and rather inefficient by both modes, and the odds of early-phase transmission was positively associated with the number of infected fleas that fed. Disease progression in individual mice bitten by fleas infected with a bioluminescent strain of *Y*. *pestis* was tracked. An early prominent focus of infection at the intradermal flea bite site and dissemination to the draining lymph node(s) soon thereafter were common features, but unlike what has been observed in intradermal injection models, this did not invariably lead to further systemic spread and terminal disease. Several of these mice resolved the infection without progression to terminal sepsis and developed an immune response to *Y*. *pestis*, particularly those that received an intermediate number of early-phase flea bites. Furthermore, two distinct types of terminal disease were noted: the stereotypical rapid onset terminal disease within four days, or a prolonged onset preceded by an extended, fluctuating infection of the lymph nodes before eventual systemic dissemination. For both modes of transmission, bubonic plague rather than primary septicemic plague was the predominant disease outcome. The results will help to inform mathematical models of flea-borne plague dynamics used to predict the relative contribution of the two transmission modes to epizootic outbreaks that erupt periodically from the normal enzootic background state.

## Author summary

*Yersinia pestis* can be transmitted by fleas within a few days after taking a blood meal from a highly bacteremic host, termed early-phase or mass transmission; and again after it forms a dense biofilm in the foregut of its vector that can eventually block blood feeding. The relative importance of the two transmission modes in the ecology of plague is a matter of current debate, but estimates of transmission rate, efficiency, and other parameters are limited. We compared transmission and disease progression dynamics in mice bitten by groups of fleas three days after their infectious blood meal (early-phase or mass transmission mode) and in mice bitten by individual blocked fleas. In general, a higher percentage of transmissions by blocked fleas led to terminal disease, whereas early-phase transmissions more often led to survival and an immune response, which are nonproductive infections in the sense that the bacteremia required to continue the *Y*. *pestis* life cycle did not develop and these animals would be removed from the pool of susceptibles in the host population. The data will be useful in mathematical models of plague dynamics in wild rodent populations.

## Introduction

*Yersinia pestis*, the bacterial agent of plague, has adapted a flea-borne transmission life cycle primarily involving rodents and several different fleas that parasitize them. The ecology of plague is complex, with different degrees of susceptibility among mammalian hosts and different degrees of vectorial capacity among different flea species. Plague is enzootic, and often unnoticed, in many areas of the world but explosive epizootics occur periodically in highly susceptible rodent populations for reasons that are not completely understood.

Transmission by flea bite is a major component of the disease dynamics and can occur by two related mechanisms. After acquiring *Y*. *pestis* in an infectious blood meal on a host with high-level bacteremia (10^8^ to 10^9^ CFU/ml), fleas have the ability to transmit it one to a few days later in their next blood meal on a naïve host. This mode of transmission was originally referred to as mass transmission, because it is not seen unless 5 to 10 or more infected fleas feed simultaneously on a naïve host, but has been more recently termed early-phase transmission [1,2]. A second phase of transmission is potentiated after *Y*. *pestis* grows in the form of a cohesive biofilm in the proventriculus, a valve between the esophagus and midgut of the flea, eventually filling the proventriculus and blocking the incoming flow of blood when the flea attempts to feed. Partial or complete blockage results in regurgitative transmission of bacteria from the proventriculus into the bite [3,4]. Complete blockage can take up to 1 to 2 weeks or longer to develop, depending on the number of bacteria ingested in the infectious blood meal and the feeding frequency of the flea, among other factors. Several lines of evidence indicate that the mechanism of early-phase transmission is also regurgitative, resulting from heavy initial bacterial colonization of the proventriculus [2,5,6].

The relative contribution of the two transmission modes in different natural flea-rodent cycles and in enzootic and epizootic scenarios has been reconsidered recently, and early-phase transmission has been proposed as an important driver of rapidly expanding epizootics [1,7]. However, it is recognized that more standardized studies to generate reliable comparative, quantitative data are needed [8]. In this study we compared transmission efficiencies and disease outcomes following early-phase and blocked flea transmission, using the North American ground squirrel flea *Oropsylla montana*. To monitor disease progression and outcome, we challenged mice with fleas infected with bioluminescent *Y*. *pestis*. This allowed a second goal of the study, which was to compare infection dynamics following the natural flea-borne route of infection to previous studies that used needle-injection of bioluminescent *Y*. *pestis* grown in culture media. The results of this study are directly informative about the infection dynamics of flea-borne plague, and potentially of greater use in informing transmission models in real-world settings.

## Results

### Early-phase transmission efficiency and outcomes

To assess early-phase transmission dynamics, groups of 4 to 16 *O*. *montana* fleas were allowed to feed on naive mice 3 days after their infectious blood meal. This represented their first feeding opportunity after infection, when early-phase transmission is at peak efficiency [2,7,9]. A total of 48 mice in ten independent experiments that were fed upon by 2 to 12 infected fleas were monitored (S1 Table). Because the host blood source used for the infectious blood meal has been shown to influence early-phase transmission frequency [5], rat blood was used for the infectious blood meal in five experiments (25 mice challenged with these fleas) and mouse blood for the other five experiments (23 mice challenged). The first three experiments were with fleas infected with wild-type *Y*. *pestis* and the remainder with fleas infected with the bioluminescent *Y*. *pestis* strain. Three basic outcomes resulted from these challenges: (I) acute terminal disease, characterized by systemic dissemination and sepsis; (II) transmission without systemic dissemination, in which infection was limited to the dermal flea bite site and usually the draining lymph node(s) and sometimes the liver and spleen, with the mice remaining asymptomatic but transmission and infection verified by bioluminescence and seroconversion; and (III) no evidence of transmission (Table 1).

**Table 1:** Outcomes of flea bite challenges.

Outcome		EPT (mouse blood)	EPT (rat blood)	EPT (cumulative)	Blocked (rat blood)
IA	Terminal disease (rapid onset)	1/23 (4%)	6/25 (24%)	7/48 (15%)	9/28 (32%)
IB	Terminal disease (prolonged onset)	2/23 (9%)	2/25 (8%)	4/48 (8%)	1/28 (4%)
IA + IB	Terminal disease (total)	3/23 (13%)	8/25 (32%)	11/48 (23%)	10/28 (36%)
II	Survival (seroconversion)	10/23 (43%)	8/25 (32%)	18/48 (37%)	3/28 (11%)
(IA + IB) + II	Transmission (total)	13/23 (57%)	16/25 (64%)	29/48 (60%)	13/28 (46%)
III	No transmission	10/23 (43%)	9/25 (36%)	19/48 (40%)	17/28 (54%)
II + III	“nonproductive” challenge	20/23 (87%)	17/25 (68%)	37/48 (77%)	18/28 (64%)

The number of mice with each outcome divided by the total number of mice challenged (%) is listed. EPT, early-phase transmission; Blocked, transmission by blocked flea bite.

**Fig 1:**
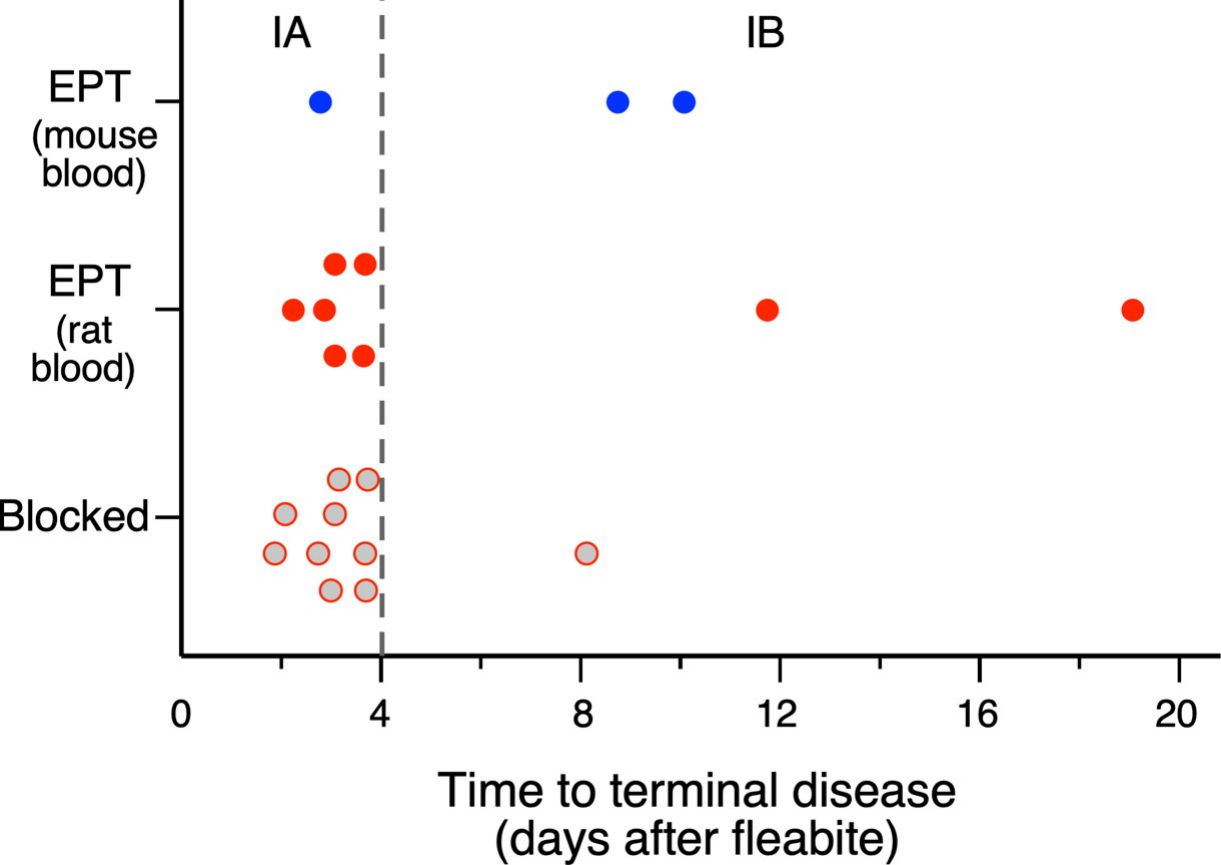
Distribution of rapid onset (within four days; IA) and prolonged onset (IB) terminal bubonic plague in mice following early-phase transmission (EPT) by fleas infected using mouse blood or rat blood, or following transmission by blocked fleas. Symbols represent individual mice.

**Fig 2:**
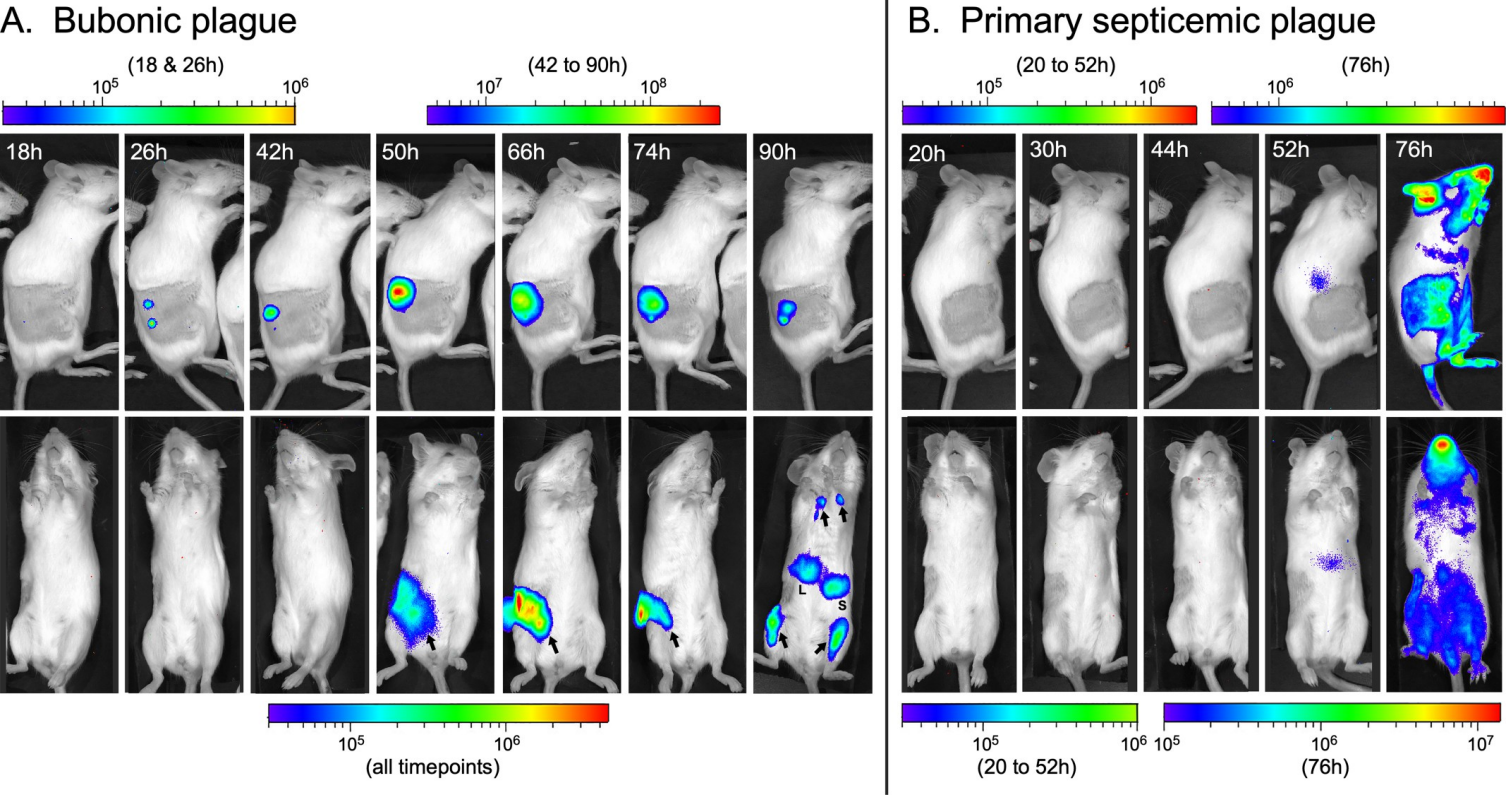
Examples of rapid onset terminal bubonic plague (**A**) and primary septicemic plague (**B**) within 4 days following flea bite. In bubonic plague, bioluminescent signal from the dermis at the flea bite sites appeared within 2 days after challenge (lateral images, top row) and increased in intensity over the following days, indicating bacterial multiplication. Dissemination to the draining lymph node was visible within 3 days (ventral images, bottom row) and systemic spread and terminal disease a day after that, within 4 days. Arrows indicate signal from lymph nodes; L = liver, S = spleen. In primary septicemic plague, no infection at the flea bite site or draining lymph node was seen; bioluminescent signal in peripheral areas appeared suddenly on day 3 together with signs of terminal disease. The scale bars indicate the intensity of bioluminescent signal (photons/sec/cm^2^/sr). Different scales were used due to the fluctuations in signal intensity throughout the observation period.

**Fig 3:**
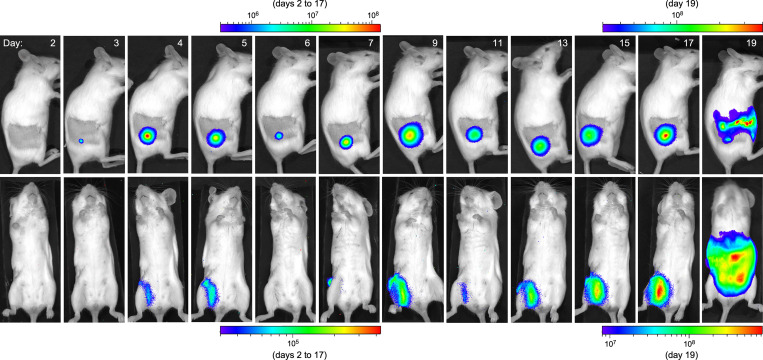
Prolonged onset terminal bubonic plague in a mouse following flea bite. A permanent focus of infection was detected by day 3 and dissemination to the draining lymph node on day 4–5. The lymph node bioluminescent signal receded and resurged twice before the infection eventually disseminated systemically to produce terminal sepsis on day 19 after transmission. The scale bars indicate the intensity of bioluminescent signal (photons/sec/cm^2^/sr). Different scales were used due to the fluctuations in signal intensity throughout the observation period.

Interestingly, two distinct presentations of acute terminal disease were observed after early-phase transmission. The stereotypical rapid, fulminant bubonic plague occurred in 7 of the 11 mice that developed terminal plague, with a focus of infection apparent at the flea bite site within 2 days, dissemination to the draining lymph node within 3 days, and systemic spread and terminal sepsis within 4 days (Outcome IA; Table 1, Figs 1 and 2A and S1). In the four other mice, however, a prolonged course was observed, with terminal disease not occurring until 8 to 19 days after flea bite challenge (Outcome IB; Table 1, Figs 1 and 3 and S1). All four mice in this category showed a focus of infection at the flea bite site that appeared 1 to 3 days after challenge and persisted until terminal disease. In two of these mice, the infection appeared to be limited to the dermal flea bite site from day 1 until day 7 to 9, and then rapidly disseminated to the draining inguinal and axillary lymph nodes and systemically to produce terminal sepsis. The two other mice showed dissemination from the skin to the draining inguinal lymph node after 3 days, and in one of them further dissemination to the ipsilateral axillary lymph node on day 7. The infection appeared to wax and wane somewhat in these lymph nodes over several days before eventually spreading systemically to produce terminal sepsis (Figs 3 and S1).

**Fig 4:**
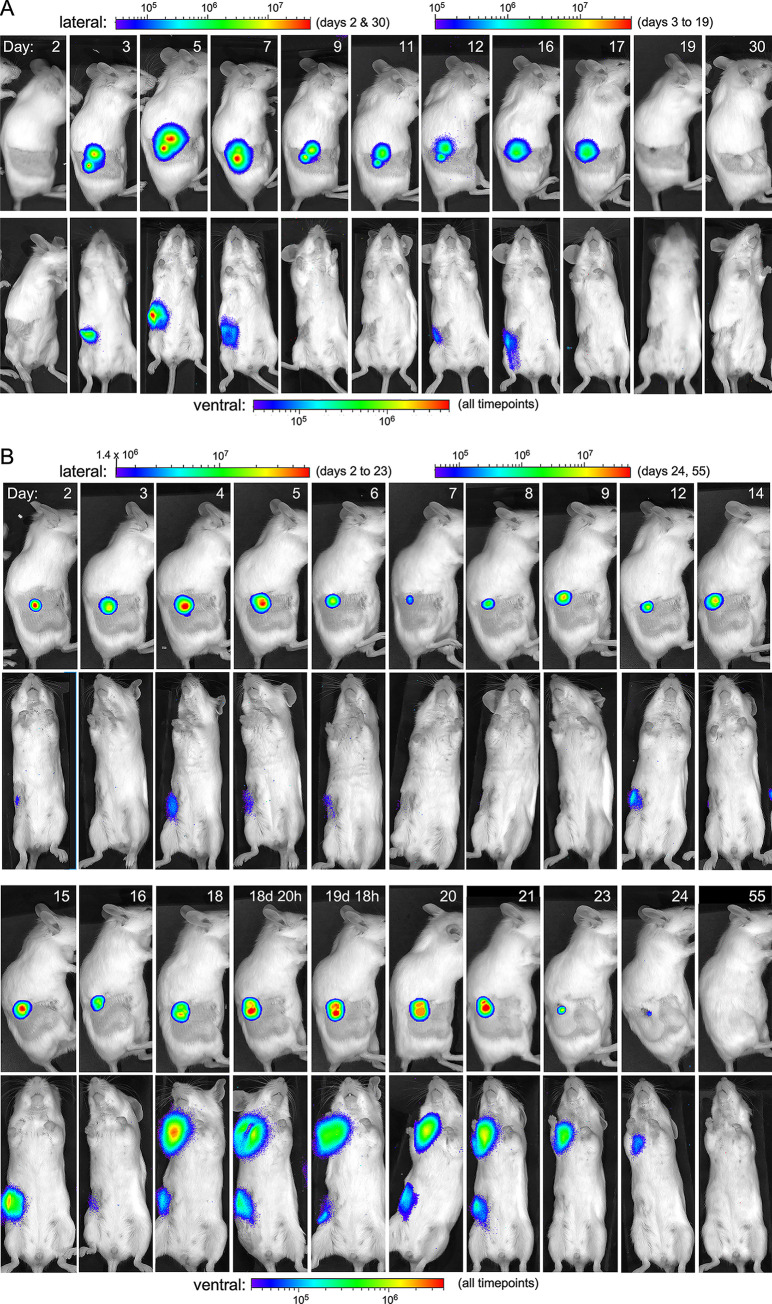
Recovery from prolonged bubonic plague infection without systemic dissemination. (**A**) Three days after early-phase transmission intradermal infection was evident at two flea bite sites and in the draining inguinal lymph node in this mouse. The infection was resolved in the skin by day 19. Infection of the draining lymph node was evident on days 3–7, but regressed on day 9 before reappearing on day 12 before finally disappearing on day 17. (**B**) Recovery from bubonic plague infection in a mouse following blocked-flea transmission. The pattern is similar to (**A**) but involves the axillary lymph node as well as the draining lymph node, and a lessening of intensity of the intradermal infection on day 7–8. Dissemination to the draining inguinal lymph node was evident on day 4 and to the ipsilateral axillary lymph node on day 18, with the intensity of infection regressing and relapsing at both sites until day 24. This mouse appeared to be completely clear of infection on day 26 and was finally euthanized on day 55. The scale bars indicate the intensity of bioluminescent signal (photons/sec/cm^2^/sr). Different scales were used due to the fluctuations in signal intensity throughout the observation period.

Ten of the 18 infected survivors (Outcome II) had been challenged by fleas infected with the bioluminescent *Y*. *pestis* strain and five of them developed IVIS-positive foci of infection. These mice showed a similar pattern as the mice with prolonged terminal outcomes, except that the infection never spread systemically (Figs 4 and S1). Dermal infections that developed at the flea bite site were detected in these mice 1 to 3 days after transmission and persisted for 9 to 30 days. Draining lymph node infections were observed beginning 5 to 10 days after transmission and persisted for 1 to 13 days but waxed and waned in intensity before disappearing (Figs 4 and S1). One mouse in this category showed only a persistent dermal infection that never disseminated. The other five surviving mice infected with the bioluminescent *Y*. *pestis* were IVIS-negative; evidently, bacterial numbers never reached detectable threshold levels in these mice and transmission was disclosed only by positive serology. Eight of the 15 mice challenged by fleas infected with wild-type (non-bioluminescent) *Y*. *pestis* were also survivors. None of these 18 Outcome II mice ever showed signs of disease and were euthanized 30 days after the early-phase flea bite challenge. They were all seropositive for *Y*. *pestis* F1 antigen, however, confirming that transmission and infection had occurred (S2 Table). Finally, 19 of the 48 mice bitten by infected early-phase fleas showed no evidence of transmission or infection by either IVIS or serology and showed no signs of disease during 30 days (Outcome III; Table 1).

### Comparison of early-phase and blocked flea transmission efficiency and outcomes

**Fig 5:**
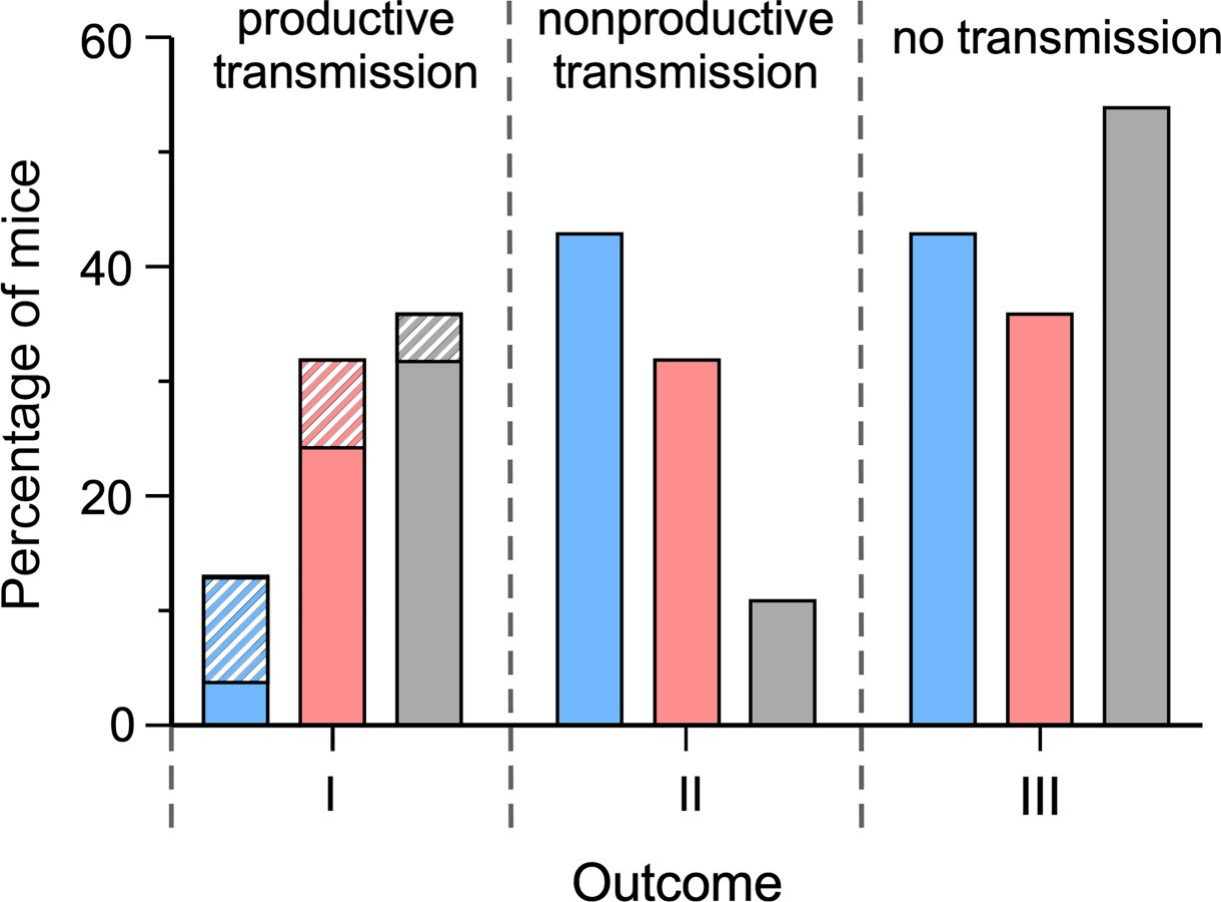
Distribution of outcomes following early-phase transmission challenges by fleas infected using mouse blood (blue bars) or rat blood (red bars); or following bites by blocked fleas (grey bars). Data are from Table 1. Productive transmission (Outcome I) is defined as terminal sepsis and bacteremia required to continue the *Y*. *pestis* life cycle; the solid portion of the bars represents the percentage of rapid onset (Outcome IA) and the hatched portion prolonged onset (Outcome IB) terminal disease. Nonproductive transmission (Outcome II) is defined as an infection that is resolved before systemic dissemination and terminal sepsis, thus precluding continuation of the flea-borne transmission cycle of *Y*. *pestis*. These mice mount an immune response and are at least temporarily removed from the pool of susceptibles in the population. No evidence of infection (no transmission; Outcome III) was detected in other mice following flea bite challenge.

In a second series of experiments, 28 mice were bitten by individual fleas that had developed proventricular blockage, 5 to 24 days after their infectious rat blood meal (S3 Table). The same three basic outcomes were seen, with challenge by individual blocked fleas leading to terminal disease (I), asymptomatic, resolved infection (II), or no evidence of transmission (III). Mice in this group had comparable rates of terminal disease (Table 1, Fig 1, Outcomes IA+IB; *p* = 1.0) and somewhat lower rates of resolved infections with seroconversion (Table 1, Outcome II; *p* = 0.19) relative to mice bitten by early-phase fleas that were similarly infected using rat blood, though neither of these comparisons was statistically significant. When compared with mice bitten by early-phase fleas, ignoring blood source used, mice bitten by fleas with proventricular blockage had slightly higher rates of terminal disease (Table 1, Outcomes IA+IB; *p* = 0.45) and lower rates of resolved infection (Table 1, Outcome II; *p* = 0.06), though, again, neither comparison was statistically significant. A visual display of the cumulative results listed in Table 1 is provided in Fig 5.

Of the 9 mice that developed rapid terminal disease following a blocked flea bite, 8 showed the typical fulminant bubonic plague that was seen following early-phase transmission (Fig 2A), but one appeared to develop primary septicemic plague, which spread directly from the flea bite site into the peripheral blood (Fig 2B). This primary septicemic form occurs occasionally if the flea transmits bacteria directly into a blood vessel instead of into the extravascular space in the dermis [10]. The infection in the one mouse with prolonged onset terminal disease had spread to the draining lymph node by day 6 but did not disseminate systemically until day 8 (S1 Fig). One of the three surviving mice with resolved infection following blocked-flea challenge (Outcome II) showed only a mildly bioluminescent focus of infection at the flea bite site that subsided by day 21 with no dissemination. In one mouse the infection had spread to the draining lymph node by day 10 but was undetectable the next day, only to reappear a few days later (similar to the pattern shown in Fig 4A) without further dissemination before appearing to die out (S1 Fig). The third mouse showed infection at the flea bite site on day 2, spread to the draining lymph node on day 15 and to the ipsilateral axillary lymph node on day 18. The intensity of infection in this mouse periodically diminished and increased at all three sites before finally disappearing on day 26 (Figs 4B and S1).

Overall, early-phase transmission efficiency (the percentage of individual fleas in the challenge group that transmit) was estimated to be 17% (95% CL 11.8–24.1%) (S1 Table). This value includes productive transmission events that led to terminal disease with high-density bacteremia (Outcome I) as well as those that led to asymptomatic, resolved limited infections and seroconversion (Outcome II), which are nonproductive in the sense that the high-density bacteremia required for the mouse-to-flea portion of the *Y*. *pestis* life cycle never develops. When only productive transmissions were considered (Outcome I), the estimated early-phase transmission efficiency was only 4.5% (95% CL 2.4–7.7%) (S1 Table). In contrast, total transmission efficiency for a blocked flea bite was 46% and productive transmission efficiency was 36% (Tables 1 and S3 and Fig 5).

The ability to relate the number of infected fleas that fed to the number of discrete intradermal foci of infection at the bite sites permitted a direct way to estimate the probability of transmission. In 35 early-phase challenges, a total of 27 IVIS-positive skin lesions developed from 199 infected flea feedings (13.6%), which matches well the calculated estimate (17%) derived statistically (S1 and S4 Tables). In contrast, 1-h exposure to 35 blocked fleas resulted in 22 IVIS-positive skin lesions among 32 mice (62.8%; S2 and S5 Tables).

### Histopathology, infection dynamics, and immune response

**Fig 6:**
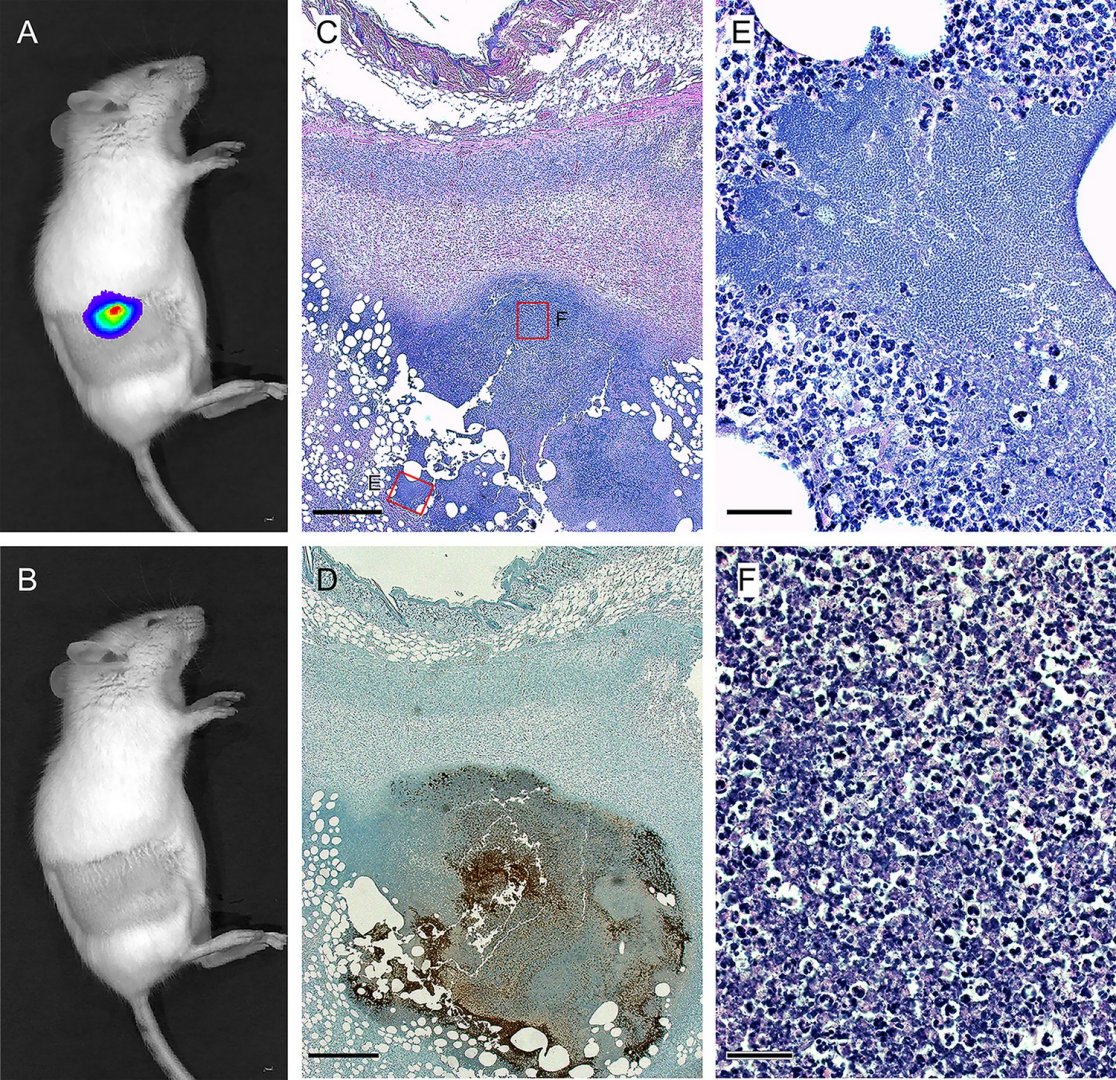
Persistent intradermal infection at the flea bite site. A mouse with terminal disease euthanized on day 10 photographed with (**A**) and without (**B**) bioluminescence detection. Skin sections taken at the site of infection were stained by H&E (**C**) and immunohistochemistry (IHC) using anti-*Y*. *pestis* antibody (**D**) and show ulcerative and necrotizing dermatitis extending into the panniculus and large numbers of bacteria (brown color on IHC). Higher magnification of areas demarcated by red boxes in (**C**) show a large dense microcolony of extracellular *Y*. *pestis* surrounded by neutrophils (**E**) and an area of viable and degenerate neutrophils and macrophages (necrosis) intermixed with *Y*. *pestis* (**F**). Scale bars = 0.5 mm (**C**, **D**); 50 μm (**E**, **F**).

The foci of active infection, as evidenced by detectable bioluminescence, that developed at the dermal flea bite sites persisted until terminal disease (8 to 18 days) for the 5 IVIS+ mice in Outcome category IB and for 9 to up to 30 days in the eight mice in category II with resolved infections (Figs 3 and 4 and S1). This was unexpected, as an active and expanding, prolonged dermal focus of infection has not been documented before. Histology of skin biopsies from prolonged terminal or resolved infections, collected upon euthanasia at the time of terminal disease or 25 to 35 days after challenge, respectively, showed evidence of extensive bacterial multiplication, with dense fields of extracellular *Y*. *pestis* still present in the IVIS+ lesions, and an immune response marked by ulcerative and necrotizing dermatitis characterized by viable and degenerate neutrophils and some macrophages and panniculitis (Fig 6, S6 Table). In some mice a black necrotic spot was evident on the surface of the skin but never appeared inflamed or swollen.

Evidence of infection was also detected in the draining lymph node, spleen, and liver of all 4 mice in Category IB and 3 of 5 mice in Category II examined, typified by multifocal necrosis, small to moderate numbers of viable and degenerate neutrophils and bacteria but few macrophages (S2 Fig, S6 Table). Large numbers of *Y*. *pestis* were also recovered from lymph node, spleen, and blood of mice with terminal disease (S2 Table).

### Effect of infectious blood source and number of infected flea bites on early-phase transmission

Previous studies have shown that *O*. *montana* infected using rat blood have higher infection rates and cumulatively transmit greater numbers of CFUs in the early phase than do fleas infected using mouse blood [5,11]. Consistent with this, we observed higher rates of both rapid onset (24% vs. 4%) and total terminal disease (32% vs. 13%) outcomes among mice bitten by fleas infected using rat blood versus mouse blood in early-phase transmission, although these differences were not statistically significant (Table 1, Figs 1 and 5; Outcome IA, *p* = 0.12; Outcomes IA+IB, *p* = 0.3) (Table 1, Figs 1 and 5). In our experiments the mice bitten by fleas infected using rat blood tended to experience greater numbers of bites (mean and median = 7) than mice exposed to fleas infected using mouse blood (mean and median = 4) (S1 Table), making the comparison more problematic.

**Fig 7:**
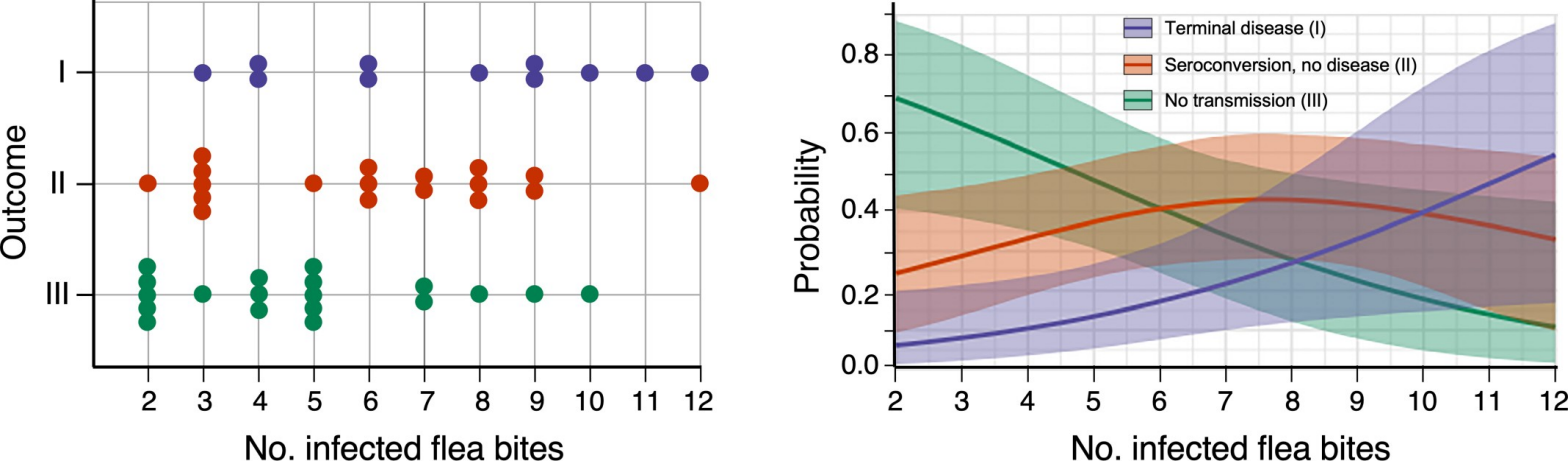
Association between the number of infected fleas that fed and early-phase transmission efficiency and disease outcome probabilities. Left panel: The outcomes of all early-phase transmission challenges (S1 Table) plotted against the number of infected fleas that fed on a mouse. Outcome I = terminal disease; Outcome II = resolved, asymptomatic infection without sepsis, resulting in seroconversion and survival; Outcome III = no transmission. Right panel: Posterior probabilities of each outcome as a function of the number of infected flea bites estimated using a Bayesian ordinal cumulative logistic regression model fit to data from the left panel. Solid lines correspond to the posterior median probabilities of each of the disease outcomes and shaded areas correspond to 95% credible intervals. See text and supplemental S1 text for details.

An overall association between transmission outcome severity and the number fleas that fed in early phase challenges was detected. We estimate that an additional flea that fed was associated with a 1.3-fold (95% CI: 1.1, 1.6) relative increase in the odds of infection or disease versus no transmission; or by proportional odds, the odds ratio of systemic dissemination versus a less severe outcome. The posterior distributions of outcome probabilities as a function of number of flea bites indicate that i) the probability that an infected mouse develops terminal disease is positively associated with the number of fed fleas; ii) the probability of no transmission is negatively associated with the number of fed fleas; and iii) mice fed upon by low to intermediate numbers of infected fleas are more likely than not to survive following transmission (Fig 7). However, there is substantial uncertainty across the range of number of fed fleas, with significant overlap in the posterior distributions of disease outcome probabilities at every level of fed fleas.

## Discussion

*Y*. *pestis* emerged as a flea-borne pathogen only within the last 6,000 years, the time frame when it diverged from *Yersinia pseudotuberculosis*, its food- and water-borne progenitor [12–15]. Perhaps reflective of a short coevolutionary history with its vector, *Y*. *pestis* is a generalist, utilizing the same simple regurgitative transmission mechanism in several different and unrelated flea species. The mechanism is based on its ability to heavily colonize the flea foregut and impede normal blood feeding. If large numbers of *Y*. *pestis* are ingested from a host with high-density bacteremia (>10^8^ CFU/ml), the bacteria rapidly localize to the proventriculus in a dense mass that can result in the early-phase transmission phenomenon [2,5,6]. The proventricular bacterial mass becomes fixed after the development of a mature biofilm and complete blockage [16,17].

Transmission by either mode appeared to be variable and rather inefficient (Table 1 and S1–S5 and Fig 5). For example, 64 to 87% of flea bite challenges did not lead to terminal disease. In the early phase, previous studies estimated a transmission efficiency of 0 to 11% when 5 to 14 *O*. *montana* infected using rat blood containing 10^8^ to 10^9^ *Y*. *pestis*/ml fed simultaneously 1 to 4 days later on highly susceptible mice, but this value included mice that seroconverted as well as those that developed terminal disease [1,18–20]. Our results are consistent with those studies, but here we also estimated the transmission efficiencies leading to these different outcomes separately and found that a significant proportion of transmissions resulted in nonproductive infections in which the terminal bacteremia necessary to complete the *Y*. *pestis* life cycle did not develop. The infection was limited to the skin and lymphatic system in these mice, and sometimes spleen and liver, and they eventually resolved it and seroconverted; thus, these mice would not be able to pass *Y*. *pestis* to uninfected fleas that feed on them. In effect, these animals are ‘vaccinated’ and removed from the pool of susceptibles in the population, a factor which should be considered in mathematical modeling of disease dynamics. Transmission following challenge by individual blocked flea bites was also variable, with an overall transmission efficiency of 46% (consistent with our previous study with *Xenopsylla cheopis* fleas [21]), but a higher proportion of these (36%) resulted in productive, terminal disease than did early-phase mass transmissions (13–32%) (Table 1, Fig 5).

In our early-phase trials, it was not possible to control the number of fleas that fed nor to know how many CFUs, if any, were transmitted in an individual bite. The number of bacteria transmitted in the early phase appeared to be highly variable, as sometimes transmission and disease was effectuated by as few as three infected fleas but in other instances 7 to 10 infected fleas that fed failed to transmit (Fig 7, S1 and S4 Tables). Compared to mouse blood, rat blood is digested more slowly and rat hemoglobin crystallizes in the flea gut, which correlates with reflux of a portion of the infected blood meal into the esophagus within 24 hours of feeding and a greater number of CFUs transmitted per flea [5]. In this study, fleas infected using rat blood tended to be more efficient early-phase transmitters than fleas infected using mouse blood (Table 1, Fig 5), suggestive that larger numbers of CFUs were transmitted by the rat blood-infected fleas, but the differences in outcome did not reach the level of statistical significance. The very low LD_50_ of the laboratory mouse (<10 CFU) makes this difference harder to discern. Presumably, the effect of infectious blood source on numbers of CFU transmitted by the early-phase mechanism would be more evident with a somewhat more resistant host. Another variable was the average number of infected fleas that fed in early-phase challenges, which by chance was higher in the rat blood experiments than in the mouse blood experiments (S1 Table). The number of CFUs transmitted by a single blocked flea bite is also known to be highly variable [21]. We hypothesize that the number of *Y*. *pestis* transmitted is directly related to disease outcome, but there is also expected to be animal-to-animal variation in immune response.

Early-phase transmission occurs upon the first blood meal after the infectious blood meal, but decreases significantly in subsequent blood meals unless the fleas are reinfected [1,9]. In contrast, blocked fleas retain their transmission efficiency while they make persistent, repeated attempts to feed during the few days before they eventually die from starvation. In our experiments, blocked fleas were given only a single one-hour feeding opportunity. Importantly, as has been noted, this underestimates the effective natural transmission efficiency of a blocked flea, which likely approaches 100% because the cumulative number of bites and extended time over which it attempts to feed act as a force multiplier for transmission [22,23]. For example, in one case a single blocked flea transmitted to three separate bite sites during the 1-hour challenge period (S5 Table). Early-phase fleas feed normally, in that they are able to take a complete blood meal within a few minutes. Thus, in the case of early-phase fleas we can be fairly confident that the number of fleas that fed is equivalent to the number of bites received.

Bubonic plague is the most common disease presentation following transmission by a blocked flea bite, but primary septicemic plague can less frequently result [10,24]. Normally, the flea mouthparts are thought to cannulate a blood vessel to feed, but blocked flea feeding differs in that they probe the dermis repeatedly in futile attempts to acquire a blood meal, conceivably promoting intradermal instead of intravascular deposition of bacteria. Because the feeding behavior of early-phase fleas appears to be more normal, like that of uninfected fleas, another objective of this study was to determine whether early-phase transmission leads to a higher incidence of primary septicemic plague. This was not the case, as the only instance of primary septicemia we observed followed transmission by a blocked flea. This result is consistent with the observation that fleas are capable of feeding from a pool of blood released from capillaries punctured during probing as well as feeding directly from a cleanly cannulated blood vessel [25,26]. Thus, intradermal transmission is the norm for both the early-phase and the blocked flea modes, indicating that regurgitation of bacteria occurs mainly during probing or feeding on extravascular blood, before intravascular blood-feeding commences.

Three previous studies have followed the progression of bubonic plague in mice following intradermal [27,28] or subcutaneous [29] injection of bioluminescent *Y*. *pestis*. Our results using the natural flea-borne route of transmission show some differences to these studies. Intradermal injection led only to the rapidly progressing, acute form of bubonic plague with little bacterial proliferation in the skin, and systemic dissemination and terminal disease within 4 days. Persistent infection of the linea alba at the subcutaneous injection site was noted in one study [29], but once the bacteria had spread to the draining lymph nodes systemic dissemination and terminal disease ensued rapidly. In contrast, extensive bacterial multiplication and a persistent, prolonged focus of infection always occurred in the dermis after flea-borne transmission (Figs 2–4 and 6). Dissemination to the draining lymph nodes also often led to an extended battle with the host immune response at those sites, where the infection could either eventually be contained or to disseminate to the spleen and liver, whereupon terminal sepsis usually occurred rapidly. Thus, as has long been supposed, the dermis and the draining lymph nodes represent the two significant bottlenecks to bubonic plague progression. It is not clear if the waxing and waning pattern of bioluminescence in the lymph node was due to fluctuations in the number of viable bacteria or in their physiological state that affect production or stability of the bioluminescent proteins. At any rate, a persistent intradermal reservoir of masses of extracellular *Y*. *pestis* represents an ongoing threat for dissemination long after transmission. Thus, mice challenged by flea bite should be monitored for at least a month to identify all cases of productive transmission.

Three mice that resolved infection without terminal disease had evidence of spleen or liver involvement (S6 Table, S2 Fig). Necrotic areas and chronic abscesses in the spleen and liver have been noted before in rats and ground squirrels and this condition without obvious disease has been termed resolving, latent, or subacute plague [30–32]. Several factors could account for differences in outcomes seen with needle-injection and flea-borne transmission models. *Y*. *pestis* has a unique phenotype in the flea that cannot be duplicated in laboratory media cultures used for injection [33]. The flea mouthparts are much smaller than an intradermal injection needle and often small numbers of bacteria are transmitted, along with flea saliva, to a specific intradermal location. The bacteria regurgitated into the dermis are also associated with digestion products of flea blood meals [2,17]. All of these factors may influence nascent infection and immunity, including the initial response by neutrophils [34], and account for the differences in pathogenesis between flea-borne and needle-injection models.

The relative inefficiency of flea-borne transmission and the large variation in numbers of bacteria transmitted are likely to be important determinants of plague ecology. In this study, we used an optimized model consisting of a highly competent vector and highly susceptible laboratory mice. Different flea vector species exhibit varying degrees of vector competence for *Y*. *pestis*, and their hosts varying degrees of susceptibility to overt disease [8]. Thus, transmission efficiencies and the ratio of disease outcomes likely vary between different flea-host cycle systems, depending on flea vector competence, characteristics of the host blood and the LD_50_ of *Y*. *pestis* for a particular host species. For so virulent a pathogen, a fine balance between terminal and immunizing infections may be critical because the coexistence of resistant and susceptible hosts in a population is thought to play a role in stable enzootic maintenance [35,36]. Due to its inherent inefficiency, a high flea burden on a highly susceptible host population is probably necessary for early-phase transmission to instigate and drive epizootics. As mentioned previously, the per-bite inefficiency of blocked-flea transmission is compensated for by the altered feeding behavior of serial feeding attempts over an extended period before the flea dies of starvation. The average number of *Y*. *pestis* transmitted by blocked *O*. *montana* fleas is also significantly higher than the number transmitted in the early phase [5,37]. This would seem to make the biofilm-dependent proventricular blockage mode of transmission a more reliable means of producing the terminal bacteremia required to complete the *Y*. *pestis* life cycle, particularly in the many natural hosts for which the LD_50_ of *Y*. *pestis* is greater than that of highly susceptible laboratory mice.

## Materials and methods

### Ethics statement

Experiments involving animals were approved by the Rocky Mountain Laboratories, National Institute of Allergy and Infectious Diseases, National Institutes of Health Animal Care and Use Committee (Animal Protocol #2015–094) and were conducted in accordance with all National Institutes of Health guidelines.

### Bacteria

The virulent *Y*. *pestis* strain 195/P [38] was used in all experiments. For *in vivo* imaging experiments, 195/P was transformed by electroporation with the bioluminescence plasmid pGEN-*lux*CDABE (Addgene plasmid # 44918) [39].

### Mice

Specific-pathogen-free, 6- to 10-week-old female Rocky Mountain Lab Swiss-Webster (RML) mice were used for all experiments.

### Flea infections

*O*. *montana* fleas from a laboratory colony [37] were infected by allowing them to feed for one hour on heparinized RML mouse blood or defibrinated Sprague-Dawley rat blood (Bioreclamation, Jericho, NY) containing 6 × 10^8^ to 2 × 10^9^ *Y*. *pestis* CFU/ml using an artificial feeder system standard protocol [37,40]. Fleas that took a full infectious bloodmeal were held at 22°C, 75% relative humidity. For blocked-flea transmission experiments, fleas were infected using rat blood, fed twice-weekly on neonatal mice for 1 hour and then examined microscopically for evidence of proventricular blockage (fresh red blood only in the esophagus that was blocked from entering the midgut) [16,37,40].

### Flea bite challenges

Early-phase transmission was evaluated by allowing groups of 4 to 16 fleas to feed on naïve mice three days after their infectious blood meal. Fleas were placed in a modified 10-ml syringe from which the end had been removed and a 100 μm mesh screen attached. After fleas were added the syringe plunger was reinserted and the mesh surface was applied for 1 hour to an area on the side of the mouse abdomen that had been shaved 1–2 days earlier. Fleas were then collected, anesthetized with CO_2_ and examined by light microscopy for evidence of feeding. The number of fleas that fed on each mouse was recorded and these fleas were frozen at -80°C for later determination of bacterial load by plating dilutions of individual triturated fleas [40]. Transmission by blocked fleas was evaluated by allowing 1 or 2 blocked fleas to feed on naïve mice beginning the day after their initial blockage diagnosis. After a 1-hour feeding opportunity, fleas were examined microscopically to determine whether they had attempted to feed and were still blocked.

### Transmission and disease monitoring

Mice were monitored three times a day for the first seven days and twice a day thereafter. When signs of terminal disease developed (lethargy, ruffled fur, hunched posture, reluctance to respond to outside stimuli) the mice were euthanized and the time recorded. From mice with rapid onset of terminal disease (euthanized within 4 days after challenge) the draining inguinal lymph node and spleen were taken for bacterial load determinations. In mice with a prolonged time to terminal disease (> 8 days after challenge), blood and spleen were collected for bacterial load. The draining inguinal lymph node and a liver sample were preserved in 10% neutral buffered formalin (10NBF) for histological analysis. These mice also had visible skin lesions, and a skin biopsy of the lesion was also preserved in 10NBF. Mice surviving to 25–30 days after challenge and asymptomatic were euthanized and the spleen was collected to measure bacterial load and the draining inguinal lymph node and a liver sample were preserved in 10NBF. If a visible skin lesion was present at any time during the experiment in these mice, a skin biopsy was preserved in 10NBF. Serum was collected from all mice surviving past 8 days for detection of antibody to *Y*. *pestis* as described below. Bacterial load in organs was enumerated by limiting dilution on blood agar plates.

### *In vivo* bioluminescent imaging

Mice bitten by fleas infected with the *Y*. *pestis* (pGEN-*lux*CDABE) strain were transferred daily from their home cages to the XIC-3 Animal Isolation Chamber (Perkin-Elmer, Waltham, MA) in a biosafety cabinet, then moved to the IVIS Lumina imaging instrument (Perkin Elmer, Caliper Life Sciences). Mice were sedated with 2.5% isoflurane and imaged both on the flea exposure area (lateral thigh) and from the ventral aspect for a maximum of 5 minutes. Images were analyzed using Living Image software v. 3.0 (Caliper Life Sciences).

To quantitate the bioluminescent signal in mice from foci of infection in the skin, lymph nodes, and other tissues, regions of interest (ROI) were defined and radiance measured in photons/sec/cm^2^/steradian (p/s/cm^2^/sr), using Living Image software v 3.2 (Perkin Elmer, Caliper Life Sciences). An ROI from an uninfected area was also measured to determine background radiance, which was subtracted from the signal.

### Detection of immune response to *Y*. *pestis*

Seroconversion of challenged mice was determined by measuring IgG and IgM responses to the F1 capsular antigen of *Y*. *pestis* using a quantitative ELISA. High protein-binding 96-well flat bottom plates (Thermo Fisher Scientific, Waltham, MA) were coated overnight at 4°C with purified recombinant F1 antigen [41] at 0.5 μg/ml in 0.05 M carbonate/bicarbonate buffer, pH 9.6. Plates were blocked with 5% powdered skim milk in PBS-0.05% Tween-20 for 2 hours at 28°C. Mouse sera were tested in triplicate at 1:100 dilution in PBS-0.05% Tween with 1% skim milk. Naïve RML mouse serum at 1:100 served as a negative control. After washing with PBS-Tween, wells were probed with either goat anti-mouse IgG (diluted 1:25,000) or goat anti-mouse IgM-horseradish peroxidase conjugate (Thermo Fisher Scientific) diluted 1:12,000 in PBS-Tween with 1% skim milk. After washing with PBS-Tween, 100 μl Ultra TMB-ELISA horseradish peroxidase substrate (Thermo Fisher Scientific) was added to the wells, and 5 minutes later 50 μl of 2 M H_2_SO_4_ fixative before absorbance (A_450_) readings on a Synergy 2 microplate reader (Winooski, VT). To quantitate antibody responses based on A_450_ readings, a standard curve was built using serial 2-fold dilutions (1:1250–1:2560K; 3 to 4 replicates per dilution) of a mouse IgG1 monoclonal antibody against F1 (clone YPF19, Thermo Fisher Scientific). The 1:2500 dilution was arbitrarily assigned a value of 10,000 antibody units (U). A standard curve of log_10_ (U) plotted against A_450_ was fitted to the four-parameter logistic regression model in Prism v.7.01 (GraphPad Software, Inc., La Jolla, CA), with the ‘hill slope’ constrained to 1.0 and the ‘bottom’ parameter set to the mean A_450_ of the negative controls. The log_10_ (U) of unknown sera was interpolated from the standard curve; mice were considered seropositive with an A_450_ greater than 2 SD above the mean negative control value.

### Histology

Tissues fixed in 10NBF were processed using a VIP-6 Tissue Tek (Sakura Finetek, USA) and embedded in Ultraffin paraffin polymer (Cancer Diagnostics, Durham, NC). Samples were sectioned at 5 μm, and resulting slides were stained with hematoxylin and eosin. Additional sections were processed for immunohistochemistry. Bacteria were detected using a polyclonal rabbit anti-*Y*. *pestis* serum at a 1:4000 dilution [17]. The secondary antibody was a prediluted biotinylated anti-rabbit antibody (Biogenex, Fremont, CA). Immunohistochemical staining was performed using the Discovery Ultra automated staining platform (Ventana Medical Systems, Tucson, AZ). Sections were evaluated by a board-certified veterinary pathologist (D. Scott) and assigned a score of 0 (normal tissue) to 4, with increasing score indicating increasing severity of pathological changes.

### Statistical analyses

Early-phase transmission efficiency, defined as the proportion of flea bites resulting in transmission, was calculated using a maximum likelihood algorithm (PooledInfRate v. 4.0) [42] comparing the number of infected fleas feeding on a mouse to transmission outcome (terminal disease, asymptomatic infection with seroconversion, or no transmission). We tested whether transmission outcomes were independent of infectious blood meal source (mouse vs. rat) and transmission modality (early-phase vs. blocked flea) via a Chi-squared test for independence without a continuity correction and with *p*-values based on 1 million Monte Carlo samples drawn under the null distribution. These analyses were performed using R version 3.6.3 [43].

To estimate the association between number of infected fleas fed and survival outcomes in mice (which are naturally ordered by severity, with no transmission being least severe, followed by survival of infected mice, and then fatal systemic infection), we fit a Bayesian ordinal cumulative logistic regression model, also referred to as a proportional odds model, for outcome versus the number of infected fleas that fed on a mouse. Across all levels of outcome severity, the model expresses the change in the log odds that the outcome for a mouse will be as, or less, severe than a given severity level as a linear function of the number of fed fleas and the blood source used to infect the fleas. The model was fit under weakly informative priors using the brms package in R [44]. Details regarding model specification, fitting, and diagnostics are provided in the supplemental S1 Text.

## Supporting information

S1 Fig Progression of *Y*. *pestis* infection in mice that developed terminal disease following flea-borne transmission. The intensity of bioluminescence (average radiance) at the flea bite site (skin) and in different tissues during the course of infection was determined by region of interest (ROI) quantitation in 26 mice in which transmission of the *Y*. *pestis* (pGEN-*lux*CDABE) strain occurred. Individual graph labels identify the mouse (E = early-phase transmission, S1 Table experiments 4–10; B = blocked-flea transmission, S3 Table). The mice used for figures in the main text are indicated.
(TIF)

S2 Fig Progression of *Y*. *pestis* infection in mice that remained asymptomatic and resolved the infection following flea-borne transmission. The intensity of bioluminescence (average radiance) at the flea bite site (skin) and in different tissues during the course of infection was determined by region of interest (ROI) quantitation in 26 mice in which transmission of the *Y*. *pestis* (pGEN-*lux*CDABE) strain occurred. Individual graph labels identify the mouse (E = early-phase transmission, S1 Table experiments 4–10; B = blocked-flea transmission, S3 Table). The mice used for figures in the main text are indicated.
(TIF)

S3 Fig Sections of spleen from a surviving, asymptomatic mouse euthanized 29 days after transmission by flea bite. Dense extracellular clusters of *Y*. *pestis* surrounded by intact and degenerating neutrophils are indicated by the arrowheads (left panel; H&E stain). Immunohistochemistry stain (right panel) in which *Y*. *pestis* stains brown. Scale bars = 50 μm.
(TIF)

S1 Table Early-phase transmission of *Y*. *pestis* by *O*. *montana*. (DOCX)

S2 Table Outcomes of early-phase transmission of *Y*. *pestis* by *O*. *montana*. (DOCX)

S3 Table Transmission of *Y*. *pestis* by blocked *O*. *montana* fleas. (DOCX)

S4 Table Correlation between number of infected fleas that fed during early-phase transmission challenges and the number of intradermal foci of infection subsequently observed. (DOCX)

S5 Table Correlation between number of blocked fleas that fed during 1h challenges and the number of intradermal foci of infection subsequently observed. (DOCX)

S6 Table Histology results summary. (DOCX)

S1 Text Modeling early-phase transmission efficiency and disease outcomes. (DOCX)

## References

[1] Eisen RJ, Bearden SW, Wilder AP, Montenieri JA, Antolin MF, Gage KL. Early-phase transmission of *Yersinia pestis* by unblocked fleas as a mechanism explaining rapidly spreading plague epizootics. Proc Natl Acad Sci USA. 2006;103:15380–5. 10.1073/pnas.0606831103 17032761 PMC1592641

[2] Hinnebusch BJ, Jarrett CO, Bland DM. "Fleaing" the plague: Adaptations of *Yersinia pestis* to its insect vector that lead to transmission. Annu Rev Microbiol. 2017;71:215–32. 10.1146/annurev-micro-090816-093521 28886687

[3] Bacot AW, Martin CJ. Observations on the mechanism of the transmission of plague by fleas. J Hygiene Plague Suppl 3 1914;13:423–39. 20474555 PMC2167459

[4] Bacot AW. Further notes on the mechanism of the transmission of plague by fleas. J Hygiene Plague Suppl 4. 1915;14:774–6. 20474604 PMC2206743

[5] Bland DM, Jarrett CO, Bosio CF, Hinnebusch BJ. Infectious blood source alters early foregut infection and regurgitative transmission of *Yersinia pestis* by rodent fleas. PLoS Pathog. 2018;14(1):e1006859 10.1371/journal.ppat.1006859 29357385 PMC5794196

[6] Dewitte A, Bouvenot T, Pierre F, Ricard I, Pradel E, Barois N, et al A refined model of how *Yersinia pestis* produces a transmissible infection in its flea vector. PLoS Pathog. 2020;16(4):e1008440 10.1371/journal.ppat.1008440 32294143 PMC7185726

[7] Wilder AP, Eisen RJ, Bearden SW, Montenieri JA, Gage KL, Antolin MF. *Oropsylla hirsuta* (Siphonaptera: Ceratophyllidae) can support plague epizootics in black-tailed prairie dogs (*Cynomys ludovicianus*) by early-phase transmission of *Yersinia pestis*. Vector Borne Zoonotic Dis. 2008;8:359–67. 10.1089/vbz.2007.0181 18454591

[8] Eisen RJ, Eisen L, Gage KL. Studies of vector competency and efficiency of North American fleas for *Yersinia pestis*: state of the field and future research needs. J Med Entomol. 2009;46:737–44. 10.1603/033.046.0403 19645275

[9] Eisen RJ, Lowell JL, Montenieri JA, Bearden SW, Gage KL. Temporal dynamics of early-phase transmission of *Yersinia pestis* by unblocked fleas: secondary infectious feeds prolong efficient transmission by *Oropsylla montana* (Siphonaptera: Ceratophyllidae). J Med Entomol. 2007;44:672–7. 10.1603/0022-2585(2007)44[672:tdoeto]2.0.co;2 17695024

[10] Sebbane F, Jarrett CO, Gardner D, Long D, Hinnebusch BJ. Role of the *Yersinia pestis* plasminogen activator in the incidence of distinct septicemic and bubonic forms of flea-borne plague. Proc Natl Acad Sci USA. 2006;103:5526–30. 10.1073/pnas.0509544103 16567636 PMC1414629

[11] Eisen RJ, Vetter SM, Holmes JL, Bearden SW, Montenieri JA, Gage KL. Source of host blood affects prevalence of infection and bacterial loads of *Yersinia pestis* in fleas. J Med Entomol. 2008;45:933–8. 10.1603/0022-2585(2008)45[933:sohbap]2.0.co;2 18826038

[12] Achtman M, Zurth K, Morelli G, Torrea G, Guiyoule A, Carniel E. *Yersinia pestis*, the cause of plague, is a recently emerged clone of *Yersinia pseudotuberculosis*. Proc Natl Acad Sci USA. 1999;96:14043–8. 10.1073/pnas.96.24.14043 10570195 PMC24187

[13] Cui Y, Yu C, Yan Y, Li D, Li Y, Jombart T, et al Historical variations in mutation rate in an epidemic pathogen, *Yersinia pestis*. Proc Natl Acad Sci U S A. 2013;110:577–82. 10.1073/pnas.1205750110 23271803 PMC3545753

[14] Rasmussen S, Allentoft ME, Nielsen K, Orlando L, Sikora M, Sjogren KG, et al Early divergent strains of *Yersinia pestis* in Eurasia 5,000 years ago. Cell. 2015;163:571–82. 10.1016/j.cell.2015.10.009 26496604 PMC4644222

[15] Spyrou MA, Tukhbatova RI, Wang CC, Valtuena AA, Lankapalli AK, Kondrashin VV, et al Analysis of 3800-year-old *Yersinia pestis* genomes suggests Bronze Age origin for bubonic plague. Nat Commun. 2018;9(1):2234 10.1038/s41467-018-04550-9 29884871 PMC5993720

[16] Hinnebusch BJ, Perry RD, Schwan TG. Role of the *Yersinia pestis* hemin storage (*hms*) locus in the transmission of plague by fleas. Science. 1996;273:367–70. 10.1126/science.273.5273.367 8662526

[17] Jarrett CO, Deak E, Isherwood KE, Oyston PC, Fischer ER, Whitney AR, et al Transmission of *Yersinia pestis* from an infectious biofilm in the flea vector. J Inf Dis. 2004;190:783–92. 10.1086/422695 15272407

[18] Vetter SM, Eisen RJ, Schotthoefer AM, Montenieri JA, Holmes JL, Bobrov AG, et al Biofilm formation is not required for early-phase transmission of *Yersinia pestis*. Microbiology. 2010;156:2216–25. 10.1099/mic.0.037952-0 20395271 PMC3068684

[19] Williams SK, Schotthoefer AM, Montenieri JA, Holmes JL, Vetter SM, Gage KL, et al Effects of low-temperature flea maintenance on the transmission of *Yersinia pestis* by *Oropsylla montana*. Vector Borne Zoonotic Dis. 2013;13:468–78. 10.1089/vbz.2012.1017 23590319

[20] Johnson TL, Hinnebusch BJ, Boegler KA, Graham CB, MacMillan K, Montenieri JA, et al Yersinia murine toxin is not required for early-phase transmission of *Yersinia pestis* by *Oropsylla montana* (Siphonaptera: Ceratophyllidae) or *Xenopsylla cheopis* (Siphonaptera: Pulicidae). Microbiology. 2014;160:2517–25. 10.1099/mic.0.082123-0 25187626 PMC4612360

[21] Lorange EA, Race BL, Sebbane F, Hinnebusch BJ. Poor vector competence of fleas and the evolution of hypervirulence in *Yersinia pestis*. J Inf Dis. 2005;191:1907–12. 10.1086/429931 15871125

[22] Burroughs AL. Sylvatic plague studies. The vector efficiency of nine species of fleas compared with *Xenopsylla cheopis*. J Hygiene. 1947;45:371–96. 10.1017/s0022172400014042 20475778 PMC2234840

[23] Durham DP, Casman EA. Threshold conditions for the persistence of plague transmission in urban rats. Risk Anal. 2009;29:1655–63. 10.1111/j.1539-6924.2009.01309.x 19878483

[24] Sebbane F, Jarrett C, Gardner D, Long D, Hinnebusch BJ. Role of the *Yersinia pestis* yersiniabactin iron acquisition system in the incidence of flea-borne plague. PLoS One. 2010;5(12):e14379 10.1371/journal.pone.0014379 21179420 PMC3003698

[25] Deoras PJ, Prasad RS. Feeding mechanism of Indian fleas *X*. *cheopis* (Roths) and *X*. *astia* (Roths). Ind J Med Res. 1967;55:1041–50. 5594375

[26] Deoras PS, Prasad RP. A note on the feeding mechanism of two fleas. Curr Sci. 1967;36:518–9.

[27] Gonzalez RJ, Weening EH, Frothingham R, Sempowski GD, Miller VL. Bioluminescence imaging to track bacterial dissemination of *Yersinia pestis* using different routes of infection in mice. BMC Microbiol. 2012;12:147 10.1186/1471-2180-12-147 22827851 PMC3436865

[28] Sun Y, Connor MG, Pennington JM, Lawrenz MB. Development of bioluminescent bioreporters for *in vitro* and *in vivo* tracking of *Yersinia pestis*. PLoS One. 2012;7(10):e47123 10.1371/journal.pone.0047123 23071730 PMC3469486

[29] Nham T, Filali S, Danne C, Derbise A, Carniel E. Imaging of bubonic plague dynamics by in vivo tracking of bioluminescent *Yersinia pestis*. PLoS One. 2012;7(4):e34714 10.1371/journal.pone.0034714 22496846 PMC3320629

[30] McCoy GW. Studies upon plague in ground squirrels. Publ Hlth Bull. 1911;43:3–51.

[31] Lister Institute Advisory Committee. Chronic or resolving plague. J Hygiene. 1912;12(Suppl):266–86. PMC2167444 20474514

[32] Pollitzer R. Plague. Geneva: World Health Organization; 1954.

[33] Chouikha I, Sturdevant DE, Jarrett C, Sun YC, Hinnebusch BJ. Differential gene expression patterns of *Yersinia pestis* and *Yersinia pseudotuberculosis* during infection and biofilm formation in the flea digestive tract. mSystems. 2019;4(1). Epub 2019/02/26. 10.1128/mSystems.00217-18 30801031 PMC6381227

[34] Shannon JG, Bosio CF, Hinnebusch BJ. Dermal neutrophil, macrophage and dendritic cell responses to *Yersinia pestis* transmitted by fleas. PLoS Pathog. 2015;11(3):e1004734 10.1371/journal.ppat.1004734 25781984 PMC4363629

[35] Wimsatt J, Biggins DE. A review of plague persistence with special emphasis on fleas. J Vector Borne Dis. 2009;46:85–99. 19502688

[36] Gascuel F, Choisy M, Duplantier JM, Debarre F, Brouat C. Host resistance, population structure and the long-term persistence of bubonic plague: contributions of a modelling approach in the Malagasy focus. PLoS Comput Biol. 2013;9(5):e1003039 10.1371/journal.pcbi.1003039 23675291 PMC3649974

[37] Hinnebusch BJ, Bland DM, Bosio CF, Jarrett CO. Comparative ability of *Oropsylla montana* and *Xenopsylla cheopis* fleas to transmit *Yersinia pestis* by two different mechanisms. PLoS Negl Trop Dis. 2017;11(1):e0005276 10.1371/journal.pntd.0005276 28081130 PMC5230758

[38] Chen TH, Foster LE, Meyer KF. Experimental comparison of the immunogenicity of antigens in the residue of ultrasonated avirulent *Pasteurella pestis* with a vaccine prepared with killed virulent whole organisms. J Immunol. 1961;87:64–71. 13692772

[39] Lane MC, Alteri CJ, Smith SN, Mobley HL. Expression of flagella is coincident with uropathogenic *Escherichia coli* ascension to the upper urinary tract. Proc Natl Acad Sci U S A. 2007;104:16669–74. 10.1073/pnas.0607898104 17925449 PMC2034267

[40] Bland DM, Brown LD, Jarrett CO, Hinnebusch BJ Macaluso K. R. Methods in Flea Research. BEI Resources. 2017;https://www.beiresources.org/Catalog/VectorResources.aspx.

[41] Andrews GP, Heath DG, Anderson GWJ, Welkos SL, Friedlander AM. Fraction 1 capsular antigen (F1) purification from *Yersinia pestis* CO92 and from an *Escherichia coli* recombinant strain and efficacy against lethal plague challenge. Infect Immun. 1996;64:2180–7. 10.1128/IAI.64.6.2180-2187.1996 8675324 PMC174053

[42] Biggerstaff BJ. PooledInfRate, Version 4.0: a Microsoft Office Excel Add-In to compute prevalence estimates from pooled samples. 2009; CDC, Fort Collins, CO.

[43] R Core Team. R: A language and environment for statistical computing. Vienna, Austria: R Foundation for Statistical Computing; 2019.

[44] Bürkner P-C. bmrs: An R package for Bayesian multilevel models using Stan. J Statistical Software. 2017;80(1):1–28.

