# Supplementary figures and images for: Comparison of the transmission efficiency and plague progression dynamics associated with two mechanisms by which fleas transmit Yersinia pestis
Source: PLoS Pathog. 2020 Dec 7;16(12):e1009092. doi: 10.1371/journal.ppat.1009092 (PMC7746306; doi:10.1371/journal.ppat.1009092)

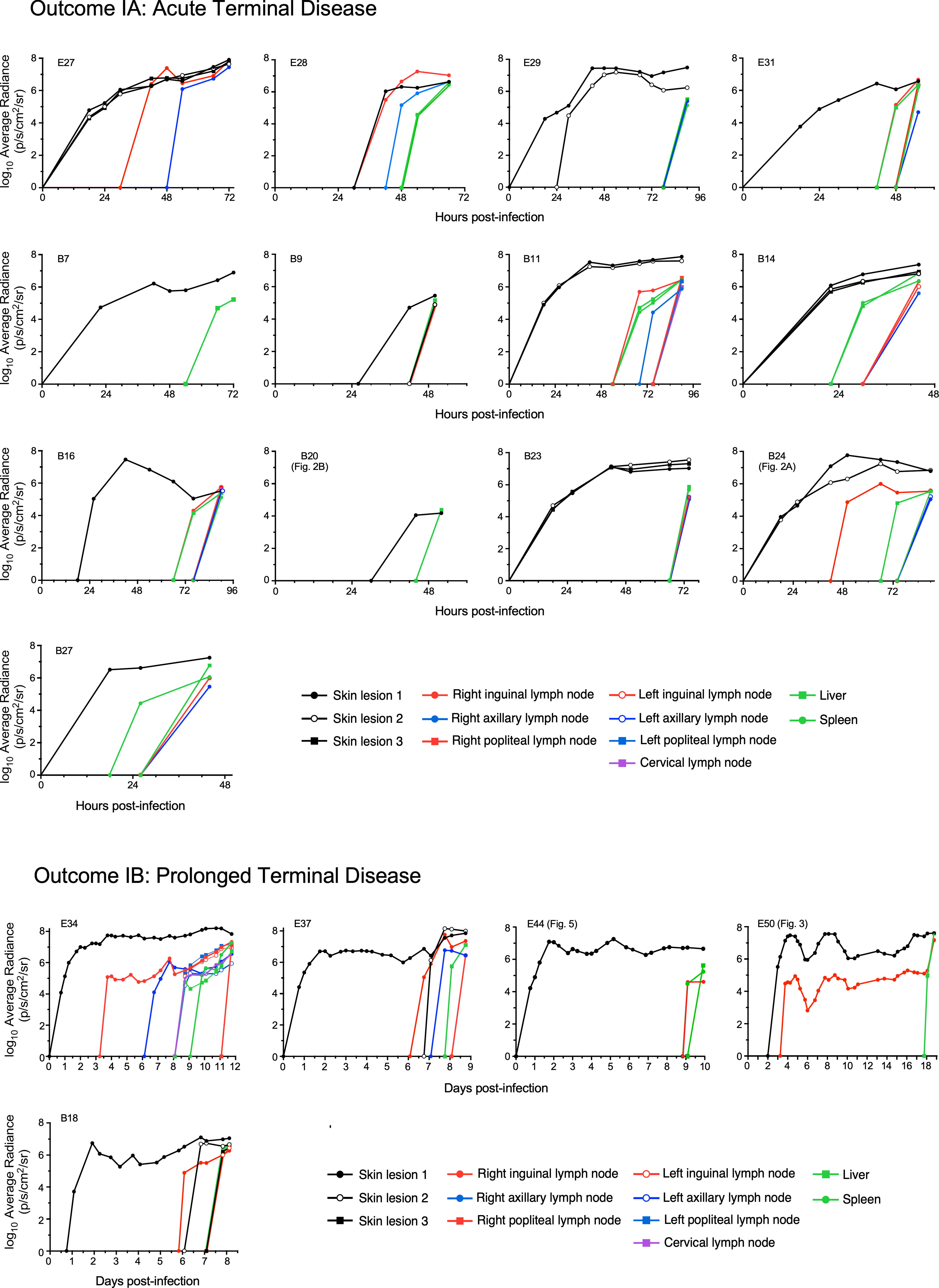

Supplement: S1 Fig — The intensity of bioluminescence (average radiance) at the flea bite site (skin) and in different tissues during the course of infection was determined by region of interest (ROI) quantitation in 26 mice in which transmission of the Y. pestis (pGEN-luxCDABE) strain occurred. Individual graph labels identify the mouse (E = early-phase transmission, S1 Table experiments 4–10; B = blocked-flea transmission, S3 Table). The mice used for figures in the main text are indicated. (TIF) [file ppat.1009092.s001.tif]

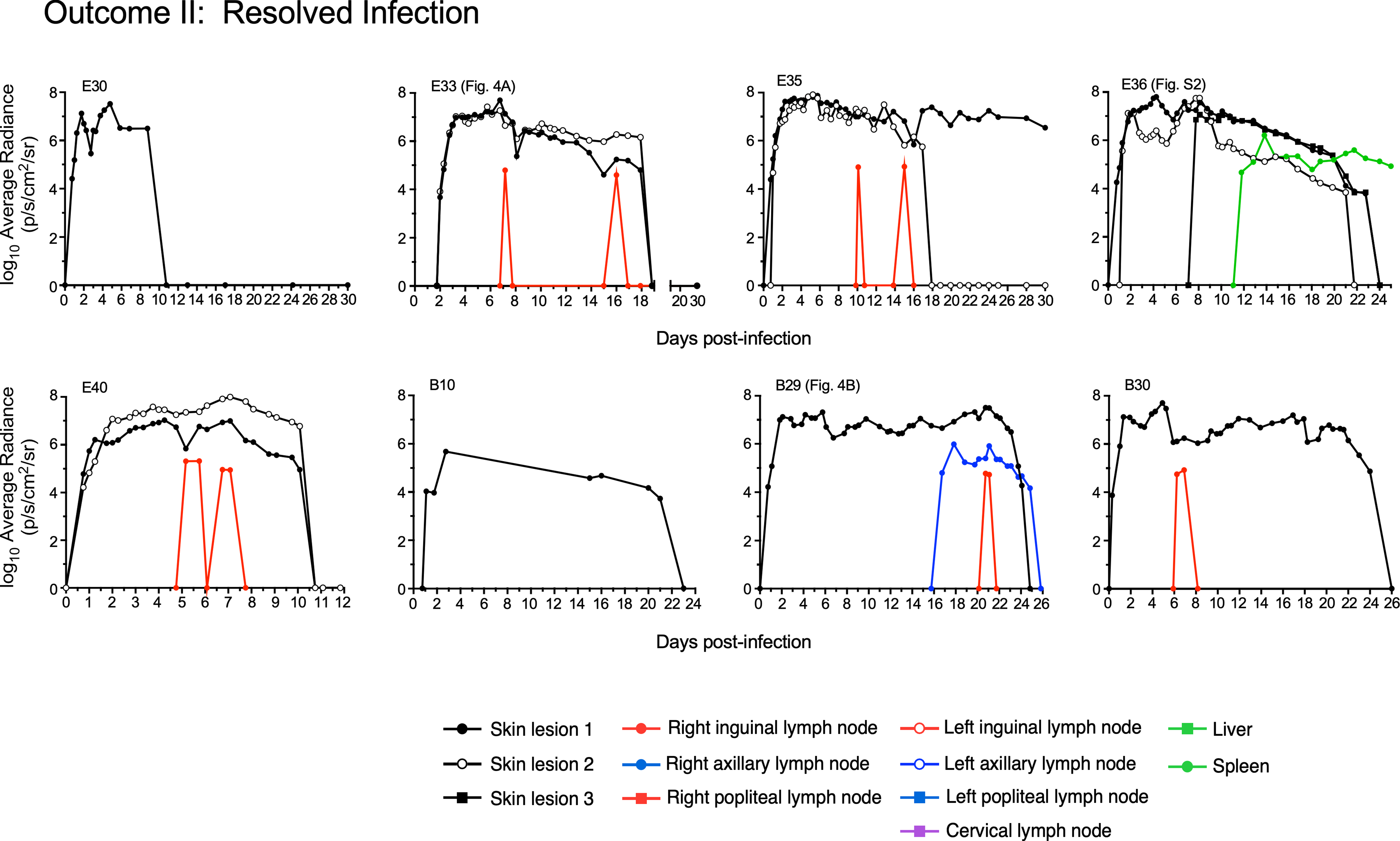

Supplement: S2 Fig — The intensity of bioluminescence (average radiance) at the flea bite site (skin) and in different tissues during the course of infection was determined by region of interest (ROI) quantitation in 26 mice in which transmission of the Y. pestis (pGEN-luxCDABE) strain occurred. Individual graph labels identify the mouse (E = early-phase transmission, S1 Table experiments 4–10; B = blocked-flea transmission, S3 Table). The mice used for figures in the main text are indicated. (TIF) [file ppat.1009092.s002.tif]

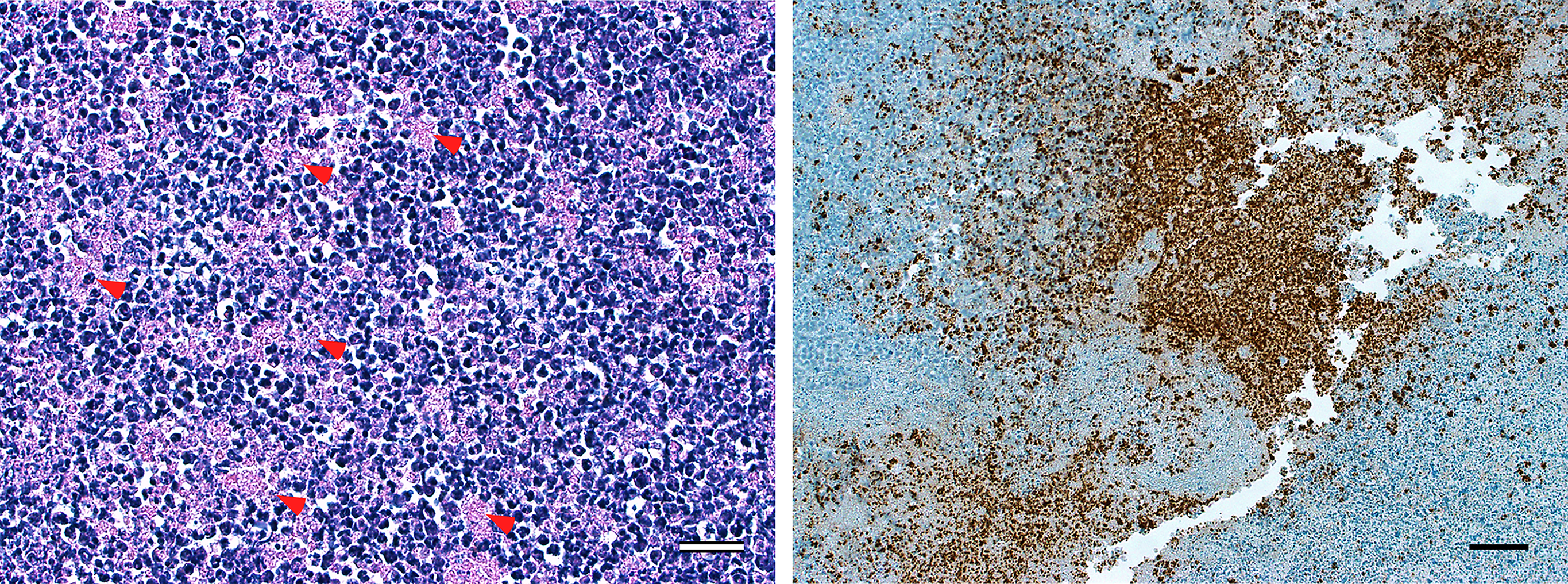

Supplement: S3 Fig — Dense extracellular clusters of Y. pestis surrounded by intact and degenerating neutrophils are indicated by the arrowheads (left panel; H&E stain). Immunohistochemistry stain (right panel) in which Y. pestis stains brown. Scale bars = 50 μm. (TIF) [file ppat.1009092.s003.tif]
