# Supplementary material for: Comparison of the transmission efficiency and plague progression dynamics associated with two mechanisms by which fleas transmit Yersinia pestis
Source: PLoS Pathog. 2020 Dec 7;16(12):e1009092. doi: 10.1371/journal.ppat.1009092 (PMC7746306; doi:10.1371/journal.ppat.1009092)
Supplement: S1 Table — (DOCX) [file ppat.1009092.s004.docx]

| **Table S1.** Early-phase transmission of *Y. pestis* by *O. montana.*  The bloodmeal titer and blood source used to infect fleas for each experiment is listed along with estimated transmission efficiency. For individual challenged mice, the number of infected fleas that fed and their bacterial loads are presented, along with the transmission outcome. | | | | | | | | |
| --- | --- | --- | --- | --- | --- | --- | --- | --- |
| Expt | *Y. pestis* CFU/ml in infectious blood meal | Mouse | No. infected fleas fed | Median (range) log CFU/fed flea | Outcome*^a^* | Transmission Efficiency (95% CI) | | |
|  |  |  |  |  |  | Outcome I | Outcome II | Total (I + II) |
| 1 | 1.6 × 10^9^ | E1 | 6 | 6.0 (5.7-6.2) | II | 7.0 | 11.4 | 11.6 |
|  | (rat blood) | E2 | 6 | 5.3 (4.6-6.3) | IA | (1.4-22.1) | (2.4-42.7) | (3.2-31.2) |
|  |  | E3 | 7 | 5.8 (5.5-6.4) | III |  |  |  |
|  |  | E4 | 7 | 6.1 (5.3-6.3) | II |  |  |  |
|  |  | E5 | 4 | 5.9 (5.5-6.1) | IA |  |  |  |
| 2 | 2.8 × 10^9^ | E6 | 3 | 5.2 (3.8-5.7) | II | 6.2 | 22.8 | 32.4 |
|  | (mouse blood) | E7 | 3 | 4.8 (4.7-5.6) | II | (0.4-27.4) | (8.7-66.5) | (12.4-69.0) |
|  |  | E8 | 4 | 5.1 (4.8-6.5) | III |  |  |  |
|  |  | E9 | 3 | 4.7 (3.9-6.4) | IA |  |  |  |
|  |  | E10 | 3 | 5.6 (5.2-6.2) | II |  |  |  |
| 3 | 1.8 × 10^9^ | E11 | 9 | 6.4 (3.1-7.0) | II | 0 | 11.0 | 6.3 |
|  | (rat blood) | E12 | 7 | 6.4 (5.8-7.1) | III |  | (3.1-32.5) | (1.2-21.3) |
|  |  | E13 | 8 | 6.0 (2.8-6.9) | II |  |  |  |
|  |  | E14 | 7 | 6.1 (2.0-6.6) | II |  |  |  |
|  |  | E15 | 5 | 6.7 (6.0-6.9) | III |  |  |  |
| 4 | 5.7 × 10^8^ | E16 | 2 | 6.1 (5.8-6.3) | II | 0 | 11.5 | 11.5 |
|  | (mouse blood) | E17 | 4 | 5.6 (5.2-5.9) | III |  | (0.8-48.2) | (0.8-48.2) |
|  |  | E18 | 2 | 5.4 (5.1-5.7) | III |  |  |  |
| 5 | 8.6 × 10^8^ | E21 | 5 | 4.4 (2.3-5.5) | III | 0 | 4.9 | 4.9 |
|  | (mouse blood) | E22 | 2 | 4.7 (4.4-4.9) | III |  | (0.3-23.5) | (0.3-23.5) |
|  |  | E23 | 6 | 5.5 (2.8-6.2) | II |  |  |  |
|  |  | E24 | 5 | 5.3 (2.3-6.1) | III |  |  |  |
|  |  | E25 | 3 | 5.7 (5.4-5.8) | III |  |  |  |
| 6 | 2.1 × 10^9^ | E26 | 10 | 6.3 (5.0-7.0) | III | 7.7 | 4.8 | 11.8 |
|  | (rat blood) | E27 | 9 | 6.1 (5.5-6.7) | IA | (2.2-22.9) | (0.4-29.0) | (4.3-35.7) |
|  |  | E28 | 12 | 5.9 (3.1-6.6) | IA |  |  |  |
|  |  | E29 | 10 | 5.9 (2.6-6.7) | IA |  |  |  |
|  |  | E30 | 9 | 5.9 (4.6-6.7) | II |  |  |  |
| 7 | 2.8 × 10^9^ | E31 | 8 | 5.7 (5.6-6.2) | IA | 4.7 | 10.8 | 13.1 |
|  | (rat blood) | E32 | 8 | 5.4 (4.7-6.2) | III | (0.9-16.0) | (3.2-39.1) | (4.7-40.9) |
|  |  | E33 | 8 | 5.9 (5.5-6.5) | II |  |  |  |
|  |  | E34 | 11 | 6.0 (5.3-6.9) | IB |  |  |  |
|  |  | E35 | 12 | 5.8 (4.8-6.7) | II |  |  |  |
| 8 | 1.9 × 10^9^ | E36 | 3 | 5.6 (5.4-5.9) | II | 5.9 | 22.0 | 36.7 |
|  | (mouse blood) | E37 | 4 | 5.8 (5.5-6.8) | IB | (0.4-26.9) | (6.4-54.8) | (13.1-84.8) |
|  |  | E38 | 2 | 3.3 (3.2-3.5) | III |  |  |  |
|  |  | E39 | 5 | 5.7 (2.9-6.3) | II |  |  |  |
|  |  | E40 | 3 | 5.7 (2.0-5.9) | II |  |  |  |
| 9 | 1.5 × 10^9^ | E41 | 6 | 5.2 (4.2-5.7) | II | 2.8 | 10.6 | 10.6 |
|  | (mouse blood) | E42 | 4 | 5.6 (5.1-5.8) | III | (0.2-14.0) | (3.0-30.7) | (3.1-30.7) |
|  |  | E43 | 9 | 5.3 (2.0-6.3) | III |  |  |  |
|  |  | E44 | 9 | 4.7 (2.5-6.3) | IB |  |  |  |
|  |  | E45 | 8 | 5.3 (2.3-5.8) | II |  |  |  |
| 10 | 1.98 × 10^9^ | E46 | 5 | 5.3 (5.2-7.0) | III | 4.9 | 0 | 4.9 |
|  | (rat blood) | E47 | 2 | 5.3 (4.6-5.6) | III |  |  |  |
|  |  | E48 | 5 | 5.5 (4.7-6.8) | III |  |  |  |
|  |  | E49 | 2 | 6.4 (6.3-6.5) | III |  |  |  |
|  |  | E50 | 6 | 5.9 (5.4-6.3) | IB |  |  |  |
|  |  |  |  | Summary: | rat blood  expts | 5.1  (2.4-9.7) | 9.1  (4.5-16.9) | 13.8  (8.3-22.4) |
|  |  |  |  | mouse blood  expts | | 3.2  (0.9-8.6) | 15.0  (8.3-25.2) | 17.9  (10.5-28.7) |
|  |  |  |  |  | all expts | 4.5  (2.4-7.7) | 11.8  (7.6-17.8) | 17.0  (11.8-24.1) |
| *^a^*Outcomes: IA = terminal disease, rapid onset (54 to 92 h after fleabite); IB = terminal disease, prolonged onset (210 to 458 h after fleabite); II = no terminal disease, transmission diagnosed by seroconversion and IVIS; III = no evidence of transmission (IVIS-negative, seronegative one month after fleabite challenge). Fleas in experiments 1-3 were infected with *Y. pestis* 195/P; fleas in experiments 4-10 were infected with *Y. pestis* 195/P (pGEN-*lux*CDABE). | | | | | | | | |
