# Supplementary material for: Comparison of the transmission efficiency and plague progression dynamics associated with two mechanisms by which fleas transmit Yersinia pestis
Source: PLoS Pathog. 2020 Dec 7;16(12):e1009092. doi: 10.1371/journal.ppat.1009092 (PMC7746306; doi:10.1371/journal.ppat.1009092)
Supplement: S2 Table — (DOCX) [file ppat.1009092.s005.docx]

| **Table S2.** Outcomes of early-phase transmission of *Y. pestis* by *O. montana* fleas | | | | | | | |
| --- | --- | --- | --- | --- | --- | --- | --- |
| Outcome^a^ | Mouse | TTD | Bacterial loads^b^ | | | anti-F1 titers log[U] | |
|  |  |  | dLN | Spleen | Blood | IgG | IgM |
| IA | E2 | 54 | - | 0 | - | - | - |
| rapid onset terminal  disease | E5 | 90 | - | >10^6^ | - | - | - |
|  | E9 | 67 | 5.2 × 10^6^ | 3.6 × 10^7^ | - | - | - |
|  | E27 | 74 | 5.0 × 10^5^ | 2.6 × 10^6^ | - | - | - |
|  | E28 | 74 | 1.6 × 10^6^ | 4.3 × 10^8^ | - | - | - |
|  | E29 | 92 | <10^3^ | 1.1 × 10^6^ | - | - | - |
|  | E31 | 69 | 3.3 × 10^5^ | 4.5 × 10^8^ | - | - | - |
| IB  prolonged onset terminal  disease | E34 | 282 | 2.2 × 10^4^ | 4.9 × 10^7^ | 3.2 × 10^4^ | 2.93 | 3.27 |
|  | E37 | 210 | - | 3.2 × 10^8^ | 5.3 × 10^6^ | 0 | 0 |
|  | E44 | 242 | 4.2 × 10^2^ | 3.3 × 10^7^ | 1.9 × 10^2^ | 1.50 | 1.92 |
|  | E50 | 458 | - | 5.1 × 10^7^ | 3.3 × 10^6^ | 0 | 0 |
| II | E1 | S | - | 0 | - | 0.66 | 1.54 |
| survivor,  sero-conversion | E4 | S | - | 0 | - | 0.46 | 1.68 |
|  | E6 | S | - | 0 | - | 0.75 | 2.08 |
|  | E7 | S | - | 0 | - | 0.26 | 1.47 |
|  | E10 | S | - | 0 | - | 0 | 1.70 |
|  | E11 | S | - | 0 | - | 3.80 | 2.86 |
|  | E13 | S | - | 0 | - | 0.35 | 1.28 |
|  | E14 | S | - | 0 | - | 0.84 | 1.94 |
|  | E16 | S | - | 0 | - | 0 | 1.14 |
|  | E23 | S | - | 0 | - | 1.86 | 2.77 |
|  | E30 | S | - | 0 | - | 3.13 | 2.32 |
|  | E33 | S | - | 0 | - | 2.10 | 2.39 |
|  | E35 | S | - | 0 | - | 2.91 | 3.27 |
|  | E36 | S | - | 0 | - | 4.47 | 2.96 |
|  | E39 | S | - | 0 | - | 1.04 | 1.71 |
|  | E40 | S | - | 0 | - | 3.96 | 2.07 |
|  | E41 | S | - | 0 | - | 0.85 | 1.32 |
|  | E45 | S | - | 0 | - | 0.79 | 1.45 |
| *^a^*Outcomes: IA = terminal disease, rapid onset (54 to 92 h after fleabite); IB = terminal disease, prolonged onset (210 to 458 h after fleabite); II = no terminal disease, transmission diagnosed by seroconversion and IVIS; III = no evidence of transmission (IVIS-negative, seronegative one month after fleabite challenge). TTD, time to terminal disease (hours); S, survivor (no terminal disease)  ^b^Bacterial load per tissue or per ml (blood); dLN, draining lymph node. | | | | | | | |
