# Supplementary material for: Comparison of the transmission efficiency and plague progression dynamics associated with two mechanisms by which fleas transmit Yersinia pestis
Source: PLoS Pathog. 2020 Dec 7;16(12):e1009092. doi: 10.1371/journal.ppat.1009092 (PMC7746306; doi:10.1371/journal.ppat.1009092)
Supplement: S3 Table — (DOCX) [file ppat.1009092.s006.docx]

| **Table S3.** Transmission of *Y. pestis* by blocked *O. montana* fleas | | | | | | | | | |
| --- | --- | --- | --- | --- | --- | --- | --- | --- | --- |
| Expt | *Y. pestis* CFU/ml in infectious  blood meal | | Mouse | | No. infected fleas that fed  (status)*^a^* | | *Y. pestis*  CFU/flea | | Outcome*^b^* |
|  |  |  |  |  |  |  |  |  |  |
| 1 | 3.6 × 10^9^ | | B1 | | 1 (B) | | 9.8 × 10^5^ | | III |
|  |  | | B2 | | 2 (B) | | 6.6 × 10^5^, 7.0 × 10^5^ | | III |
|  |  | | B3 | | 1 (B) | | 6.0 × 10^5^ | | III |
| 2 | 7.5 × 10^8^ | | B4 | | 1 (B) | | 1.2 × 10^6^ | | III |
|  |  | | B5 | | 1 (B) | | 1.3 × 10^5^ | | III |
| 3 | 1.8 × 10^9^ | | B6 | | 1 B) | | 7.0 × 10^5^ | | III |
|  |  | | B7 | | 1 (B) | | 1.0 × 10^6^ | | IA |
|  |  | | B8 | | 1 (B) | | 2.6 × 10^6^ | | III |
|  |  | | B9 | | 1 (B) | | 8.0 × 10^5^ | | IA |
|  |  | | B10 | | 2 (PB) | | 1.3 × 10^5^, 7.0 × 10^5^ | | II |
|  |  | | B11 | | 2 (B) | | 5.1 × 10^5^, 3.9 × 10^5^ | | IA |
| 4 | 1.2 × 10^9^ | | B13 | | 1 (B) | | 1.1 × 10^5^ | | III |
|  |  | | B14 | | 2 (B) | | 3.1 × 10^5^, 8.0 × 10^5^ | | IA |
|  |  | | B15 | | 2 (B) | | 1.3 × 10^5^, 4.9 × 10^5^ | | III |
|  |  | | B16 | | 1 (B) | | 2.5 × 10^5^ | | IA |
|  |  | | B18 | | 1 (B) | | ND | | IB |
|  |  | | B19 | | 1 (B) | | 3.4 × 10^5^ | | III |
|  |  | | B20 | | 2 (B) | | 8.6 × 10^5^, 2.4 × 10^5^ | | IA |
| 5 | 1.0 × 10^9^ | | B21 | | 1 (B) | | 4.9 × 10^5^ | | III |
|  |  | | B22 | | 1 (B) | | 6.3 × 10^5^ | | III |
|  |  | | B23 | | 2 (B) | | 4.2 × 10^5^, 1.8 × 10^5^ | | IA |
|  |  | | B24 | | 1 (B) | | 1.0 × 10^6^ | | IA |
|  |  | | B25 | | 1 (B) | | 3.1 × 10^5^ | | III |
|  |  | | B27 | | 1 (B) | | ND | | IA |
|  |  | | B28 | | 1 (B) | | 1.2 × 10^5^ | | III |
|  |  | | B29 | | 1 (B) | | ND | | II |
|  |  | | B30 | | 1 (B) | | 2.3 × 10^5^ | | II |
|  |  | | B31 | | 1 (B) | | 2.3 × 10^5^ | | III |
| Summary  (all expts) | | Outcome I  (terminal disease) | | Outcome II  (seroconversion) | | Total  Transmission  (I + II) | | No Transmission (III) | |
|  | | 10/28 (36%) | | 3/28 (11%) | | 13/28 (46%) | | 15/28 (54%) | |
| *^a^*Status after feeding attempt B = completely blocked; PB = partially blocked  *^b^*Outcomes: IA = terminal disease, rapid onset (45 to 92 h after fleabite); IB = terminal disease, prolonged onset (195 h after fleabite); II = no terminal disease, transmission diagnosed by seroconversion and IVIS; III = no evidence of transmission (IVIS-negative, seronegative one month after fleabite challenge)  ND = not determined. Fleas in all experiments were infected with *Y. pestis* 195/P (pGEN-*lux*CDABE). | | | | | | | | | |
