# Supplementary material for: Comparison of the transmission efficiency and plague progression dynamics associated with two mechanisms by which fleas transmit Yersinia pestis
Source: PLoS Pathog. 2020 Dec 7;16(12):e1009092. doi: 10.1371/journal.ppat.1009092 (PMC7746306; doi:10.1371/journal.ppat.1009092)
Supplement: S4 Table — (DOCX) [file ppat.1009092.s007.docx]

| **Table S4.** Correlation between number of infected fleas that fed during early-phase transmission challenges and the number of intradermal foci of infection subsequently observed. | | | |
| --- | --- | --- | --- |
| **Mouse ID** | **Number of**  **infected fleas that fed** | **Number of**  **IVIS+ skin lesions** | **Outcome** |
| E16 | 2 | 0 (1)* | II |
| E17 | 4 | 0 | III |
| E18 | 2 | 0 | III |
| E21 | 5 | 0 | III |
| E22 | 2 | 0 | III |
| E23 | 6 | 0 (1)* | II |
| E24 | 5 | 0 | III |
| E25 | 3 | 0 | III |
| E26 | 10 | 0 | III |
| E27 | 9 | 3 | IA |
| E28 | 12 | 1 | IA |
| E29 | 10 | 2 | IA |
| E30 | 9 | 1 | II |
| E31 | 8 | 1 | IA |
| E32 | 8 | 0 | III |
| E33 | 8 | 2 | II |
| E34 | 11 | 1 | IB |
| E35 | 12 | 2 | II |
| E36 | 3 | 3 | II |
| E37 | 4 | 2 | IB |
| E38 | 2 | 0 | III |
| E39 | 5 | 0 (1)* | II |
| E40 | 3 | 2 | II |
| E41 | 6 | 0 (1)* | II |
| E42 | 4 | 0 | III |
| E43 | 9 | 0 | III |
| E44 | 9 | 1 | IB |
| E45 | 8 | 0 (1)* | II |
| E46 | 5 | 0 | III |
| E47 | 2 | 0 | III |
| E48 | 5 | 0 | III |
| E49 | 2 | 0 | III |
| E50 | 6 | 1 | IB |
| **Total:** | **199** | **27** |  |
| Outcomes: IA = terminal disease, rapid onset (54 to 92 h after fleabite); IB = terminal disease, prolonged onset (210 to 458 h after fleabite); II = no terminal disease, transmission diagnosed by seroconversion and IVIS; III = no evidence of transmission (IVIS-negative, seronegative one month after fleabite challenge)  *No IVIS+ skin lesions ever developed, yet transmission had occurred as evidenced by positive serology (Outcome II). Thus, at least one (1) bite had been positive for transmission. | | | |
