# Supplementary material for: Comparison of the transmission efficiency and plague progression dynamics associated with two mechanisms by which fleas transmit Yersinia pestis
Source: PLoS Pathog. 2020 Dec 7;16(12):e1009092. doi: 10.1371/journal.ppat.1009092 (PMC7746306; doi:10.1371/journal.ppat.1009092)
Supplement: S5 Table — (DOCX) [file ppat.1009092.s008.docx]

| **Table S5.** Correlation between number of blocked fleas that fed during 1h challenges and the number of intradermal foci of infection subsequently observed | | | |
| --- | --- | --- | --- |
| **Mouse ID** | **Number of blocked fleas that fed** | **Number of**  **IVIS+ skin lesions** | **Outcome** |
| B1 | 1 | 0 | III |
| B2 | 2 | 0 | III |
| B3 | 1 | 0 | III |
| B4 | 1 | 0 | III |
| B5 | 1 | 0 | III |
| B6 | 1 | 0 | III |
| B7 | 1 | 1 | IA |
| B8 | 1 | 0 | III |
| B9 | 1 | 2 | IA |
| B10 | 2 | 1 | II |
| B11 | 2 | 2 | IA |
| B13 | 1 | 0 | III |
| B14 | 2 | 3 | IA |
| B15 | 2 | 0 | III |
| B16 | 1 | 1 | IA |
| B18 | 1 | 3 | IB |
| B19 | 1 | 0 | III |
| B20 | 2 | 1 | IA |
| B21 | 1 | 0 | III |
| B22 | 1 | 0 | III |
| B23 | 2 | 3 | IA |
| B24 | 1 | 2 | IA |
| B25 | 1 | 0 | III |
| B27 | 1 | 1 | IA |
| B28 | 1 | 0 | III |
| B29 | 1 | 1 | II |
| B30 | 1 | 1 | II |
| B31 | 1 | 0 | III |
| **Total:** | **35** | **22** |  |
| Outcomes: IA = terminal disease, rapid onset (54 to 92 h after fleabite); IB = terminal disease, prolonged onset (210 to 458 h after fleabite); II = no terminal disease, transmission diagnosed by seroconversion and IVIS; III = no evidence of transmission (IVIS-negative, seronegative one month after fleabite challenge). | | | |
