# Supplementary material for: Comparison of the transmission efficiency and plague progression dynamics associated with two mechanisms by which fleas transmit Yersinia pestis
Source: PLoS Pathog. 2020 Dec 7;16(12):e1009092. doi: 10.1371/journal.ppat.1009092 (PMC7746306; doi:10.1371/journal.ppat.1009092)
Supplement: S6 Table — (DOCX) [file ppat.1009092.s009.docx]

| **Table S6**. Histology results summary | | | | | | | | | |
| --- | --- | --- | --- | --- | --- | --- | --- | --- | --- |
| Outcome: | IB | (prolonged onset terminal disease) | | | II | (survivor, positive serology) | | | |
| Mouse^a^: | E34 | E37 | E44 | E50 | E30 | E33 | E35 | E36 | E40 |
| Tissue and histopathology^b^ | Histopathology score^c^ | | | | | | | | |
| Skin |  |  |  |  |  |  |  |  |  |
| dermatitis and panniculitis, suppurative with ulceration | 4 | 3 | 4 | 4 | 0 | 0 | 3 | 4 | 0 |
| extracellular bacterial masses | 3 | 2 | 3 | 0 | 0 | 0 | 3 | 0 | 0 |
| IHC | 4 | 2 | 4 | 3 | 0 | 0 | 2 | 3 | 0 |
|  |  |  |  |  |  |  |  |  |  |
| Draining lymph node |  |  |  |  |  |  |  |  |  |
| follicular hyperplasia | - | 0 | - | 0 | - | - | 1 | 0 | 1 |
| granulomatous inflammation | - | 0 | - | 0 | - | - | 0 | 2 | 0 |
| necrotizing steatitis | - | 4 | - | 0 | - | - | 0 | 0 | 0 |
| IHC | - | 4 | - | 1 | - | - | 0 | 0 | 0 |
|  |  |  |  |  |  |  |  |  |  |
| Spleen |  |  |  |  |  |  |  |  |  |
| necrosis with numerous bacilli | 4 | - | - | - | - | - | - | 0 | - |
| abscesses | 0 | - | - | - | - | - | - | 5 | - |
| IHC | 3 | - | - | - | - | - | - | 3 | - |
|  |  |  |  |  |  |  |  |  |  |
| Liver |  |  |  |  |  |  |  |  |  |
| necrosis with acute inflammation | - | 3 | 3 | 3 | 0 | 0 | 2 | 0 | 1 |
| IHC | - | 3 | 1 | 0 | 0 | 0 | 0 | 0 | 0 |
| ^a^Mouse identification numbers; from Table S1.  ^b^IHC = immunohistochemistry with anti-*Y-pestis* antibody  ^c^Score 0 = normal, 1 = minimal, 2 = mild, 3 = moderate, 4 = marked, 5 = severe; dash indicates no sample | | | | | | | | | |
