# Supplementary material for: Comparison of the transmission efficiency and plague progression dynamics associated with two mechanisms by which fleas transmit Yersinia pestis
Source: PLoS Pathog. 2020 Dec 7;16(12):e1009092. doi: 10.1371/journal.ppat.1009092 (PMC7746306; doi:10.1371/journal.ppat.1009092)
Supplement: S1 Text — (DOCX) [file ppat.1009092.s010.docx]

**Supplemental Methods: Modeling early-phase transmission efficiency and disease outcomes**

**Model specification and fitting procedure**

We fit the following ordered cumulative logistic regression model using the brms package in R, which is built on the Stan package for performing Bayesian inference via Hamiltonian Monte Carlo [1,2].

logit(Pr(*Y_i_* ≤ *j*|β)) = β_0_,*_j_* − β_1_ × *NumFed_i_*

*Y_i_* was the observed ordered categorical outcome, coded 1, 2, or 3 for death, transmission with survival, or no transmission, respectively, and the index j denotes the severity level. *NumFed_i_* was the count of fleas fed on mouse *i*.

For all model parameters, we assigned independent student-t priors with three degrees of freedom, mean zero, and standard deviation five, which were weakly informative for the model and variable scales. The posterior distribution was sampled using five Markov chain Monte Carlo (MCMC) chains, run for 5,000 iterations per chain, with the first 2,500 iterations discarded as warmup. MCMC convergence diagnostics did not indicate that there was any evidence of lack of convergence, as all potential scale reduction factors were less than 1.00005. The minimum bulk and tail effective sample sizes among retained samples were 8,947 and 8,171, respectively. Simulations from the posterior predictive distribution, plotted below, indicated that the

model posterior adequately captured the distribution of survival outcomes. Maximum likelihood estimates, obtained using the MASS package [3] and presented in the table below, estimates did not differ substantially from the Bayesian model, which was preferred for computational reasons.


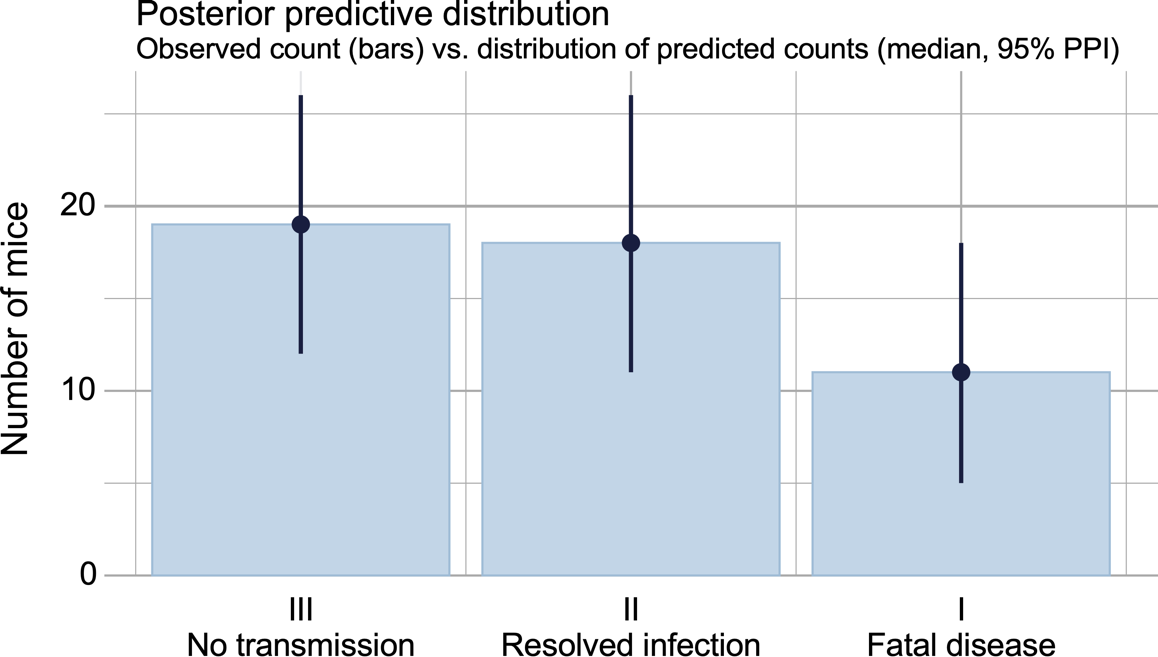


| **Table 1.** Bayesian and likelihood model estimates of proportional odds regression slop parameters | | |
| --- | --- | --- |
|  | Bayesian model | Maximum likelihood |
| NumFed | 0.3 (0.1, 0.5) | 0.3 (0.1, 0.5) |

Raw model output

## Family: cumulative

## Links: mu = logit; disc = identity

## Formula: surv − num_fleas

## Data: dat (Number of observations: 48)

## Samples: 5 chains, each with iter = 5000; warmup = 2500; thin = 1;

## total post-warmup samples = 12500

##

## Population-Level Effects:

|  | Estimate | Est. Error | 1-95% CI | u-95% CI | Rhat | Bulk_ESS | Tail_ESS |
| --- | --- | --- | --- | --- | --- | --- | --- |
| Intercept (1) | 1.05 | 0.67 | -0.26 | 2.41 | 1.00 | 9779 | 8305 |
| Intercept (2) | 2.96 | 0.79 | 1.49 | 4.60 | 1.00 | 8976 | 8854 |
| num_fleas | 0.27 | 0.11 | 0.06 | 0.48 | 1.00 | 8947 | 8171 |

## Family Specific Parameters:

|  | Estimate | Est. Error | 1-95% CI | u-95% CI | Rhat | Bulk_ESS | Tail_ESS |
| --- | --- | --- | --- | --- | --- | --- | --- |
| disc | 1.00 | 0.00 | 1.00 | 1.00 | 1.00 | 12500 | 12055 |

## Samples were drawn using sampling (NUTS). For each parameter, Bulk_ESS

## and Tail_ESS are effective sample size measures, and Rhat is the potential

## scale reduction factor on split chains (at convergence, Rhat = 1).

Maximum likelihood fit

## Call:

## polr(formula = surv − num_fleas, data = dat)

##

## Coefficients:

|  | Value | Std. Error | t value |
| --- | --- | --- | --- |
| num_fleas | 0.2543 | 0.1027 | 2.477 |

##

## Intercepts:

|  | Value | Std. Error | t value |
| --- | --- | --- | --- |
| no_transmission\|infected | 1.0034 | 0.6488 | 1.5467 |
| infected\|died | 2.8302 | 0.7663 | 3.6931 |

## Residual Deviance: 96.38382

## AIC: 102.3838

**Adjusting for blood feed type**

As a sensitivity analysis, we fit an ordinal cumulative logistic regression model, which expresses the log odds that disease severity in the *i*^th^ mouse will fall at or below the *j*^th^ severity category (denoted *Y_i_* ≤ *j*) as a linear function of the number of flea bites, the species of the infecting blood meal, and an interaction between the two allowing for the association between number of fleas fed and disease outcome to vary by species of blood meal [4].

We again fit the model using the brms package in R. Mathematically, we express

logit(Pr(*Y_i_* ≤ *j*|β)) = β_0,_ *_j_* − β_1_ × *NumFed_i_* − β_2_ × *MouseBlood_i_* − β_3_ × *NumFed_i_* × *MouseBlood_i_*

where *Y_i_* was the ordered categorical outcome, coded 1, 2, or 3 for death, transmission with survival, or no transmission, respectively. *NumFed_i_* was the count of fleas fed on mouse i. *MouseBlood_i_* was an indicator, coded 0 if the fleas fed on mouse *i* were infected using rat blood, and 1 if mouse blood. For all model parameters, we assigned independent student-t priors with three degrees of freedom, mean zero, and standard deviation five, which were weakly informative for the model and variable scales.

Among mice bitten by fleas infected with rat blood, we estimate that for two mice differing in their number of fed fleas by one, the ratio of the odds of transmission leading to fatal or resolved infections, versus no transmission is 1.5 (95% CI: 1.1, 2.1), with the mouse with more feeding fleas having the higher odds of severe outcome. By proportional odds, this is also equal to the odds ratio of fatal disease versus less severe outcome. Among mice exposed to fleas infected with mouse blood, we estimate the odds ratio of transmission leading to fatal or resolved infections versus no transmission, again also equal to the odds ratio of death versus infection or no transmission by proportional odds, to be 1.2 (95% CI: 0.5, 2.8), with the mouse with more feeding fleas having higher odds of severe outcome. We can also compute the marginal posterior probabilities, plotted below along with 95% credible intervals, of each of the three disease outcomes, which are averaged over infecting species. The probability of no transmission is negatively associated with the number of fed fleas. The probability that an infected mouse dies is positively associated with the number of fed fleas, although infected mice are more likely to survive for low to intermediate numbers of fed fleas. There is substantial uncertainty across the range of number of fed fleas, with significant overlap in the posterior distributions of disease outcome probabilities at every level of fed fleas, likely reflecting the wide range in CFUs transmitted in individual bites. A comparison of the model adjusted for blood feed species with the unadjusted model (Fig 7) on the basis of expected log predictive density indicates that the two models are comparable in their predictive performance.


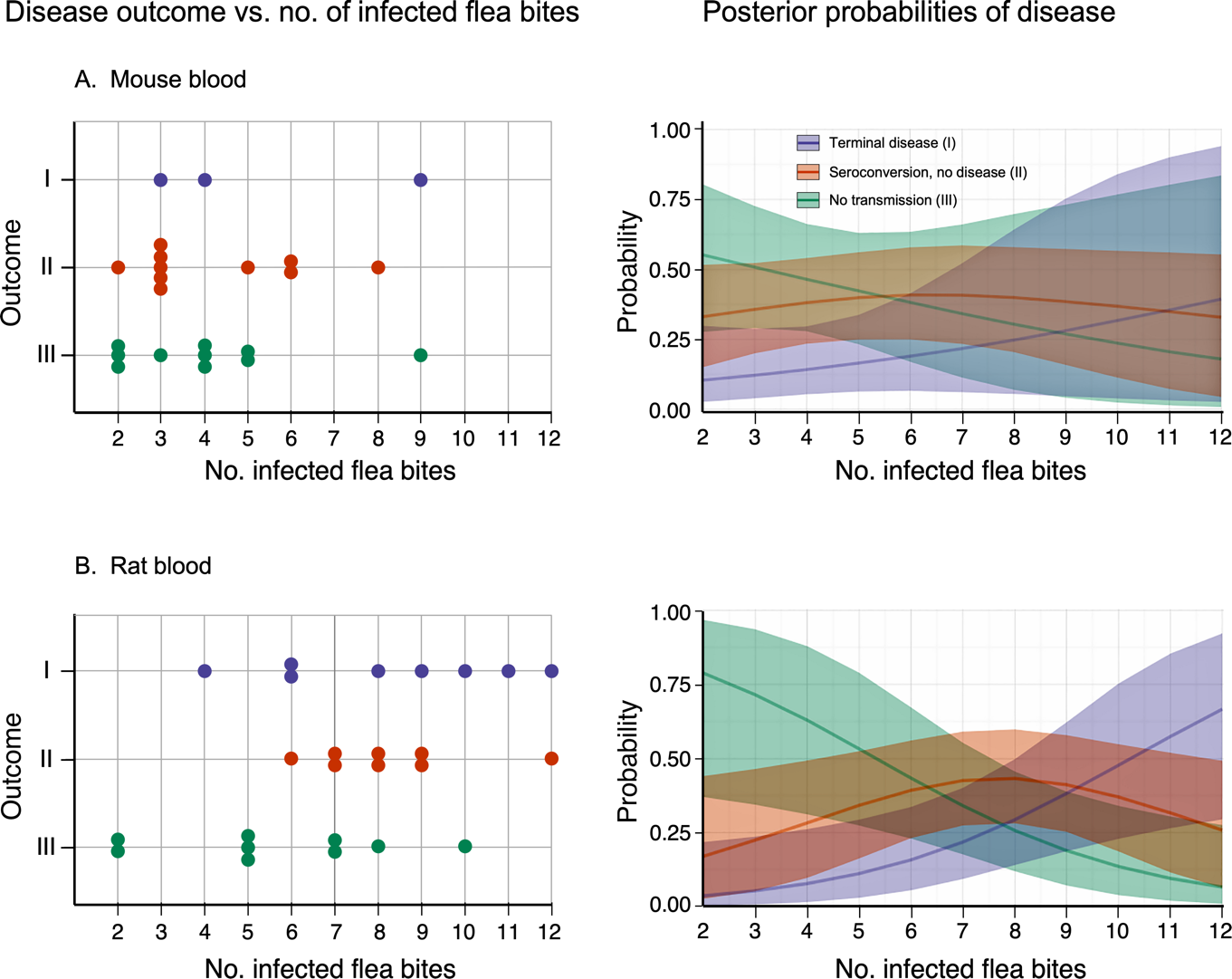


| **Table 2.** Comparison of fitted models for outcome versus number of infected flea bites with and without adjustment for infectious blood meal source. | | |
| --- | --- | --- |
|  | Unadjusted model | Adjusted for blood source |
| elpd_diff | 0.00 | -1.97 |
| se_diff | 0.00 | 1.40 |
| elpd_loo | -51.40 | -53.37 |
| se_elpd_loo | 3.04 | 3.70 |
| p_loo | 3.19 | 5.79 |
| se_p_loo | 0.33 | 0.93 |
| looic | 102.81 | 106.75 |
| se_looic | 6.08 | 7.41 |

**References**

1. Bürkner PC . brms: an R package for Bayesian multilevel models using Stan. J. Statistical Software 2017 80.1:1-28.
2. Stan Development Team. RStan: the R interface to Stan. R package version 2.17.3. 2018: <http://mc-stan.org>
3. Ripley B, Venables B, Bates DM, Hornik K, Gebhardt A, Firth D, Ripley MB. Package ‘mass’. Cran R. 2013: https://cran.r-project.org/web/packages/MASS/index.html
4. Bürkner PC, Vuorre M. Ordinal regression models in Psychology: A tutorial. Adv Meth Practices Psycholog Sci. 2018: 2515245918823199.
